# World health Organization’s guidance for tracking non-communicable diseases towards sustainable development goals 3.4: an initiative for facility-based monitoring

**DOI:** 10.1016/j.eclinm.2025.103304

**Published:** 2025-07-02

**Authors:** Arlene Quiambao, Mohammad-Reza Malekpour, Ali Golestani, Mahsa Heidari-Foroozan, Seyyed-Hadi Ghamari, Mohsen Abbasi-Kangevari, Benjamin O. Anderson, Prebo Barango, Elena Fidarova, Bianca Hemmingsen, Andre Ilbawi, Taskeen Khan, Roberta Ortiz Sequeria, Gojka Roglic, Sarah Rylance, Felipe Roitberg, Leanne Riley, Slim Slama, Lubna Bhatti, Melanie Cowan, Patricia Rarau, Stefan Savin, Farshad Farzadfar, Kouamivi Mawuenyegan Agboyibor, Ashutosh N. Aggarwal, Oyetayo Akala, Chaisiri Angkurawaranon, Hong Anh Chu, Ranjit Mohan Anjana, Carmen Antini, Zeba Aziz, Shannon Barkley, Abdul Basit, Partha Basu, Sara Benitez Majano, Kazi Bennoor, Jeffrey W. Brettler, Neslihan Cabioglu, Roberta Caixeta, Norman Campbell, Carolina Chavez, Sohel Reza Choudhury, Marilys Anne Corbex, Alvaro Cruz, Nemdia Daceney, Shona Dalal, Goodarz Danaei, Jean-Marie Dangou, Wouter De Groote, Cheick Bady Diallo, Issimouha Dille, Rolando Enrique Domingo, Gampo Dorji, Brucel B. Duncan, Uzochukwu Egere, Hicham El Berri, Asma El Sony, Mai Eltigani, Jill Farrington, Heba Fouad, Paola Friedrich, Soad Fuentes-Alabi, Angelo Gamarra, Edward Gregg, Reena Gupta, Sumit Gupta, Weiping Jia, Evelyn Jiagge, Pekka Jousilahti, Ratnasabathipillai Kesavan, Somesh Kumar, Tiina Laatikainen, Bagher Larijani, Maria Lasierra Losada, Tuyet Lan Le Thi, Naomi Levitt, Sylvana Luciani, Mauricio Maza, Bente Mikkelsen, Yousser Mohammad, Andrew Moran, Ali Motlagh, Stephen Mulupi, Raul H. Murillo, Miriam Mutebi, Rebecca Nantanda, Moffat Nyirenda, Patrick O’Connor, Dike Ojji, Gertrude Edna Omoro, Dolores Ondarsuhu, Pedro Ordunez, Mohamed Ould Sidi Mohamed, Mayowa Owolabi, Kazem Rahimi, Ivo Rakovac, Joao Filipe Raposo, Andres Rosende, Jane Rowley, Rengaswamy Sankaranarayanan, Vitaly Smelov, Josaia Tiko, Marcello Tonelli, Elena Tsoyi, Todd Tuttle, Cherian Varghese, Liliana Vásquez, Kavitha Viswanathan, Hongyi Xu, Cheng-Har Yip

**Affiliations:** a Department of Noncommunicable Diseases, Rehabilitation and Disability, WHO, Geneva, Switzerland; b Non-Communicable Diseases Research Center, Endocrinology and Metabolism Research Institute, Tehran University of Medical Sciences, Tehran, Iran; c University of Washington, Seattle, WA, USA; d Communicable and Noncommunicable Diseases Cluster, WHO Regional Office for Africa, Brazzaville, Congo; e Postgraduate Institute of Medical Education and Research, Chandigarh, India; f Chiang Mai University, Chiang Mai, Thailand; g Department of Data and Analytics, WHO, Geneva, Switzerland; h Dr Mohan’s Diabetes Specialities Centre, Chennai, India; i Department of Noncommunicable Diseases and Mental Health, WHO Regional Office for the Americas, Washington, DC, United States of America; j Hameed Latif Hospital, Lahore, Pakistan; k Special Programme on Primary Health Care, WHO, Geneva, Switzerland; l Diabetic Association of Pakistan, Karachi, Pakistan; m Early Detection, Prevention, and Infections Branch, International Agency for Research on Cancer, Lyon, France; n National Institute of Diseases of the Chest and Hospital, Dhaka, Bangladesh; o Kaiser Permanente, Los Angeles, United States of America; p Istanbul University, Istanbul, Türkiye; q University of Calgary, Calgary, Canada; r National Heart Foundation Hospital and Research Institute, Dhaka, Bangladesh; s Special Initiative on Noncommunicable Diseases and Innovation, WHO Regional Office for Europe, Copenhagen, Denmark; t Universidade Federal de Bahia, Salvador, Brazil; u Expertise France, Paris, France; v Global HIV, Hepatitis and STIs Programmes, WHO, Geneva, Switzerland; w Harvard T.H. Chan School of Public Health, Boston, United States of America; x Division of Programmes for Disease Control, WHO Regional Office for the Western Pacific, Manila, Philippines; y Division of Healthier Populations and Noncommunicable Diseases, WHO Regional Office for South-East Asia, New Delhi, India; z Universidade Federal do Rio Grande do Sul, Porto Alegre, Brazil; aa Liverpool School of Tropical Medicine, Liverpool, United Kingdom; ab Division of Noncommunicable Diseases, WHO Regional Office for the Eastern Mediterranean, Cairo, Egypt; ac Epidemiological Laboratory for Public Health Research and Development, Khartoum, Sudan; ad St Jude Children’s Research Hospital, Memphis, United States of America; ae Imperial College London, London, United Kingdom; af University of California San Francisco, San Francisco, United States of America; ag Hospital for Sick Children, Toronto, Canada; ah Shanghai Jiao Tong University Affiliated Sixth People’s Hospital, Shanghai, China; ai Henry Ford Cancer Institute, Detroit, United States of America; aj Finnish Institute for Health and Welfare (THL), Helsinki, Finland; ak Jhpiego, Baltimore, United States of America; al Institute of Public Health and Clinical Nutrition, Faculty of Medicine, University of Eastern Finland, Helsinki, Finland; am Tehran University of Medical Sciences, Tehran, Iran; an Viet Nam Respiratory Society, Hanoi, Viet Nam; ao University of Cape Town, Cape Town, South Africa; ap Latakia University, Latakia, Syria; aq Resolve to Save Lives, New York, United States of America; ar Shahid Beheshti Medical University, Tehran, Iran; as Pontificia Universidad Javeriana, Bogotá, Colombia; at Aga Khan University, Karachi, Pakistan; au Makerere University Lung Institute, Kampala, Uganda; av London School of Hygiene and Tropical Medicine, London, United Kingdom; aw HealthPartners Institute, Bloomington, United States of America; ax University of Abuja, Abuja, Nigeria; ay University of Ibadan, Ibadan, Nigeria; az University of Oxford, Oxford, United Kingdom; ba APDP-Diabetes Portugal, Lisbon, Portugal; bb Karkinos Healthcare, Bengaluru, India; bc University of Minnesota Health Clinics and Surgery Center, Minneapolis, United States of America; bd Prasanna School of Public Health, Manipal Academy of Higher Education, Manipal, India; be Consultant to the World Health Organization, Geneva, Switzerland; bf University Malaya Medical Centre, Kuala Lumpur, Malaysia

**Keywords:** Noncommunicable diseases, Health information systems, Quality indicators, Primary health care, Delphi technique, World Health Organization

## Abstract

**Background:**

Non-communicable diseases (NCDs) account for over 60% of annual global deaths, disproportionately affecting low- and middle-income countries. This trend undermines progress toward Sustainable Development Goal (SDG) 3.4, which seeks to reduce premature mortality from NCDs by one-third by 2030. Despite the availability of effective and relatively affordable interventions, addressing NCDs requires sustained, coordinated efforts and robust monitoring systems. Facility-based monitoring offers a dynamic alternative to static surveys, enabling continuous assessment of healthcare quality and utilization.

**Methods:**

This study followed a systematic approach to develop standardized global and national NCD monitoring indicators, using the Donabedian model as a conceptual framework. It focused on four major NCD categories: hypertension and cardiovascular diseases (CVDs), diabetes, chronic respiratory diseases, and cancers. The methodology included systematic scoping reviews from inception up to November 2021 and a multi-round Delphi process involving global experts to assess the validity and feasibility of proposed indicators. This study was funded internally by WHO. There were no payments to participants.

**Findings:**

The final output consisted of 81 validated indicators—22 core and 59 optional. These indicators demonstrated high feasibility and relevance for facility-based monitoring of NCD service delivery. They provide actionable metrics for assessing and improving the quality of care across diverse health system settings.

**Interpretation:**

This study highlights the urgent need for comprehensive, context-sensitive NCD monitoring frameworks. The proposed set of indicators offers a validated foundation for improving NCD care delivery and aligns with efforts to achieve SDG target 3.4. Ongoing updates and local adaptations will be essential to ensure continued relevance and effectiveness.

**Funding:**

This study was funded internally by 10.13039/100004423WHO.

**Research in context**

**Evidence before this study**

Previous efforts by the World Health Organization (WHO) have introduced technical packages such as the Package of Essential Noncommunicable Disease Interventions for Primary Health Care (PEN), the HEARTS technical package for cardiovascular disease management, and HEARTS-D for type 2 diabetes. These initiatives provided disease-specific guidelines and indicators aimed at strengthening NCD care in primary healthcare settings. However, there remained a critical gap in establishing a comprehensive, facility-based monitoring system with standardized indicators applicable across multiple NCDs. Furthermore, existing monitoring efforts were often fragmented, lacking a unified approach to data collection, indicator definition, and integration into national health information systems.

**Added value of this study**

This study presents the first globally coordinated initiative, led by the World Health Organization, to develop and validate a set of standardized facility-based indicators across seven high-burden NCDs. Through a Delphi process involving over 100 global experts and a systematic scoping review of the literature, we developed 81 indicators covering inputs, processes, and outcomes. These indicators are integrated with WHO technical packages such as HEARTS and PEN, enhancing their global applicability and scalability.

**Implications of all the available evidence**

This set of standardized NCD indicators may offer health systems, particularly in low- and middle-income countries, a validated framework for strengthening routine data collection and decision making at the facility level. The indicators are designed to be flexible, allowing integration into digital platforms and adaptation based on local capacity. If implemented, they could substantially improve service delivery monitoring, resource allocation, and tracking of progress toward SDG target 3.4.

## Introduction

Non-communicable diseases (NCDs) account for about two-third of all-cause mortality in 2021, equivalent to more than 40 million deaths at the global level. There are more than 16 million deaths among people aged 30–69 years caused by NCDs, more than 85% of which occur in low and middle-income countries.^1^ Cardiovascular diseases, cancers, chronic respiratory diseases, and diabetes are the four groups of diseases responsible for more than 80% of all NCDs mortality, which result in 17.9, 9.3, 4.1, and 1.5 million annual deaths in 2021, respectively.^1^

The Sustainable Development Goals (SDGs) recognize NCDs as a significant challenge for sustainable development, with SDG 3.4 urging the United Nations (UN) member states to reduce premature deaths from NCDs by one-third during 2015–2030.^2^ In response, the World Health Organization (WHO) has crafted technical packages encompassing standards and tools for screening, detection, treatment, and palliative care for NCDs, to help countries tackle the four groups of NCDs causing the majority of premature mortality in low- and middle-income countries.^3^, ^4^, ^5^

Nevertheless, most countries were off track in achieving SDG target 3.4 even before the COVID-19 pandemic, and NCD service integration into primary healthcare systems has been suboptimal.^6^^,^^7^ Now, with even scarcer resources and overstretched healthcare systems, countries face an urgent need to make substantial progress towards this target.^8^

The Global Action Plan for Healthy Lives and Well-being for All (SDG3 GAP) monitoring framework highlights the need to focus on locally driven processes in global health initiatives. It underscores a collaborative role for development partners, with a specific emphasis on strengthening lead ministries, especially the health ministry, for effective coordination. A key emphasis is on tailored coordination mechanisms that include subnational structures. In this sense, the framework encourages the use of local monitoring systems, stressing the importance of aligned strategies, predictable and unconditional long-term funding. Furthermore, it suggests allowing sufficient time for national governments to respond to requests, placing a significant emphasis on the monitoring aspect of health programs.^9^

To navigate this critical challenge, countries must equip themselves with concrete and timely evidence-based data at the sub-national and national levels. This data is indispensable for shaping effective policies, devising locally pertinent interventions, accelerating reductions in cause-specific mortality, and monitoring and evaluating advancements toward SDG target 3.4.^10^ Routine monitoring of NCD care cascade within primary care settings can unveil critical gaps in service coverage and quality, empowering health policymakers to make evidence-based decisions regarding healthcare service delivery. However, two fundamental aspects have often been overlooked: individual monitoring, which encompasses the routine collection, compilation, and analysis of individual-related indicators gathered either through paper forms or digital means during each visit, and program monitoring, which involves the regular tracking of priority indicators related to a program, encompassing inputs, outputs, and outcomes.

To ensure that indicators are comparable across facilities, subnational, national, and global levels, and to enable consistent chronological assessments, a unified approach is essential. This necessitates a systematic effort to establish a global consensus on a standardized set of indicators that offer a comprehensive perspective on the care cascade. This study aimed to provide a framework and a concise set of relevant, valid, and feasible standardized indicators for the four primary disease groups: hypertension and cardiovascular diseases (CVDs), diabetes, chronic respiratory diseases, and cancers. These indicators were based on the Donabedian model,^11^ with the ultimate goal of fortifying NCD monitoring by capitalizing on existing national health information systems, especially routine health facility reporting systems and health facility survey systems. To ensure the robustness of the developed indicators, this study also included a systematic scoping review to assess whether and how the proposed indicators have been incorporated into the scientific literature. This review served as a validation step for the Delphi process by aligning the selected indicators with existing research evidence.

## Methods

### Overview

In response to the need for a comprehensive framework to monitor NCD management in primary care facilities, WHO has designed this study. It proposes a streamlined set of relevant, valid, and feasible standardized indicators to guide the recording and reporting of health services data at the primary healthcare level. These indicators were developed to assess the detection, screening, treatment, complications, and programs’ outputs and outcomes of seven major NCDs including hypertension and CVDs, diabetes mellitus, chronic respiratory diseases (specifically asthma and chronic obstructive pulmonary diseases), breast cancer, cervical cancer, childhood cancers, and general cancers. Delphi method was designed to assess the validity and feasibility of the indicators. The relationship between the systematic scoping review and the Delphi process was twofold. First, the Delphi process generated a proposed set of indicators based on expert consensus. To validate these indicators, we conducted a systematic scoping review to examine whether and how they had been incorporated into the scientific literature. This review did not serve to introduce new indicators but was used to assess their relevance, feasibility, and prior usage in research and practice. The scoping review results were mapped against the Delphi outcomes, allowing us to identify areas of alignment between expert consensus and published evidence. If an indicator was strongly supported by expert opinion but had limited presence in the literature, additional discussions were held to determine its validity for inclusion. Conversely, if the literature strongly endorsed an indicator that had received mixed Delphi scores, further expert consultation helped clarify its feasibility in real-world settings.

### Developing NCD monitoring indicators

WHO established an expert group for each of seven high-burden and high-volume NCD diseases/risk factors including hypertension and CVDs, diabetes mellitus, chronic respiratory diseases (specifically asthma and chronic obstructive pulmonary diseases), breast cancer, cervical cancer, childhood cancers, and general cancers. To align the proposed indicators with global priorities, WHO technical products and guidelines, and the feasibility of collection, the experts analyzed WHO technical packages for NCDs, including package of essential noncommunicable disease interventions for primary health care (PEN),^3^ technical package for cardiovascular disease management in primary health care (HEARTS),^4^ and diagnosis and management of type 2 diabetes (HEARTS-D),^5^ to identify and extract proposed indicators systematically. Extracted indicators were then classified according to the Donabedian model,^11^ structured around three components (structure, process, and outcomes), to clearly reflect each stage of service delivery. Subsequently, each group developed a service delivery flowchart for their respective diseases, delineating the continuum of care and determining relevant individual- and facility-level indicators based on the primary health care measurement framework.^12^ The Donabedian model facilitated comprehensive assessment of the quality of care provided within health systems.

The indicators were designed to evaluate the availability of inputs comprising availability of medicines, devices, laboratory test performance, or sampling for cytopathologic tests; indicators regarding service delivery including early detection, screening, referring patients, treatment, and assessment of complications; indicators regarding outcomes including the quality of care; and indicators regarding essential aspects of healthcare delivery and data qualifications. The last set of indicators comprises the cross-cutting indicators, which are not specific to an NCDs program but address essential aspects of healthcare delivery and data qualifications and are necessary to interpret other indicators. The cross-cutting indicators contained the availability of trained staff indicator, completeness and timeliness of reporting indicator, supervisory supportive visit indicator, and loss to follow-up patients registered in the healthcare facility indicator.

Every indicator was assigned a meta-data stipulating the indicators’ definition, purpose, numerator, denominator, method of calculation, aggregation (by district, province, state, and national levels), disaggregation level (stratified by health facility, provider ownership type (public/private), and individual characteristics where possible and applicable), source of data (such as health facility patient registers, or individual records), key data elements (based on the definition of the indicator), frequency of reporting (annually, quarterly, biannually, etc.), users of data (such as facility, district, province, and state-level program managers), limitations of the indicators, and related links. While we used WHO guidelines for defining these indicators, we also suggested that countries could use their national data sources for data collection and calculation of indicators. In addition, the expert panel categorized indicators based on their priority in core and optional indicators. Core indicators are indicators that are considered vital for the NCDs program at national and sub-national levels. Optional indicators are those indicators that are important to be measured. In countries with limited resources, due to suboptimal infrastructures, low functional referral pathways, the high burden of collecting needed data, and the shortage of workforce at the facility level, the collection of needed data for core indicators is prioritized.

### Delphi survey

Following the development of indicators by WHO expert groups, a broader group of internal (affiliated to WHO) and external participants were gathered as the expert panel for Delphi process to assess the validity and feasibility of the proposed indicators in their field of expertise. Considering the paucity of consensus on the number of experts to be included in the expert panel and the recommendation of at least 30 members, we aimed to invite 50 experts from the internal group and 50 from the external experts to the panel. We identified potential panel experts by accessing professional networks and by contacting international experts in disease-specific fields including experts from academia, research institutions, healthcare settings, nongovernmental and international organizations, and WHO experts in health information systems and primary care service delivery.

On 9 February 2022, an invitation to a meeting was sent to all eligible participants through electronic mail along with documents regarding the indicators’ list and its meta-data and a link to an online survey. During the meeting, the purpose of the study and the indicators development process were summarized. Then, experts were grouped based on their field of expertise and were asked to delve deeply into related indicators. The participants must answer the questions of the online survey within 14 days. They also received a follow-up email should there be any clarifications needed.

Following the analysis of respondents’ feedback, the second round of expert group discussions was held from 1 to 4 March 2022, dedicated to each NCD group of indicators. During this round, a structured consensus-building approach was followed, where experts discussed indicators that had borderline scores or substantial variability in expert ratings. If an indicator did not initially meet the selection threshold (≥3 on a 5-point scale), but experts expressed strong support, it was re-evaluated through structured discussions and iterative voting until consensus was reached. The final decision to include or exclude such indicators was determined based on collective agreement.

After the first round of the panel meeting, the experts were asked to rate the validity and feasibility characteristics of indicators via an online survey through responding to quantitative and qualitative questions. In the quantitative part, all participants were asked to rate each indicator based on six questions. The first three questions evaluated the validity and included validity, having a clear and consistent definition, and sensitivity to performance. The second three questions evaluated feasibility and included importance to stakeholders, collectability, and simplicity or ease of interpretation. In the qualitative section, there were two fields for free commenting by panel. Participants could provide specific considerations regarding each indicator, provide additional comments on the comprehensiveness of pre-proposed indicators, and recommend any additional indicators. The source of individual feedback was kept confidential.

In the quantitative section, all six questions of each indicator were evaluated on a 5-point Likert scale as one indicating “very low” to five indicating “very high”. Participants were asked to choose one scale for each question of each indicator. The average ratings for questions were considered as the method of evaluation of validity and feasibility for each indicator. The average score of the first three questions (i.e., validity, having a clear and consistent definition, and sensitivity to performance) was considered as a validity score. The average score of the second three questions (i.e., importance to stakeholders, collectability, and simplicity or ease of interpretation) was considered as a feasibility score. Indicators with validity and feasibility scores of 3 or higher on the 5-point Likert scale were selected for inclusion. If an indicator did not meet the threshold but was strongly advocated for by experts, it was brought into a second round of discussions in expert group sessions. These discussions allowed for further refinement and decision-making regarding whether to include or exclude the indicator based on consensus. Disagreement was considered to occur if the validity and/or feasibility scores of an indicator lie within 1–2 on the five-point Likert scale.

The comments and suggestion section were qualitatively reviewed by the technical core team in WHO and were grouped into five categories: 1) comments resulted in major modifications, 2) comments resulted in no modifications, 3) comments resulted in accepting variations at national and subnational levels, 4) comments resulted in minor modifications, and 5) comments resulted in future considerations. Modifications on indicators’ title, purpose, definition, numerator, denominator, and method of calculation were considered major modifications. Modifications, which were not major were considered minor modifications. Group three of comments was a group that proposed minor modifications, which were not considered common among all countries and were specified to a determined area. In this case, a “context-specific modification” item is added to the meta-data of indicators, which will be completed at the time of indicator adoption. The comments about quality-related concerns, hospital and tertiary-level services inclusion, and expansion to private sectors were considered as the future consideration.

### Statistics

Quantitative data from the Delphi survey were analyzed using descriptive statistics. For each indicator, mean scores were calculated separately for validity (based on three criteria: overall validity, clarity of definition, and sensitivity to performance) and feasibility (based on importance to stakeholders, collectability, and ease of interpretation), each rated on a 5-point Likert scale (1 = very low to 5 = very high). Indicators with average scores ≥ 3.0 for both validity and feasibility were retained for inclusion. Measures of dispersion, including interquartile ranges (IQRs), were used to assess variability across expert ratings. Indicators with borderline scores or high variability were reviewed in a second Delphi round for further discussion and consensus. Given the ordinal nature of Likert-scale data and the qualitative purpose of the Delphi process, a consensus-based, non-parametric approach was appropriate. No inferential statistical tests were applied. All analyses were performed using the pandas library in Python 3.

### Ethics

This study, including the Delphi survey, received clearance from the Quality Assurance, Norms and Standards Department, the World Health Organization, with reference number HQ-2024-04377. All participants invited to the Delphi panel completed a declaration of interest and provided informed consent electronically prior to participation. Participants were free to participate in one or more domains based on their individual experience and field of expertise. Participation was voluntary, and responses were anonymized during analysis to ensure confidentiality.

### Systematic scoping review

We conducted a systematic scoping review of the literature in adherence with the preferred reporting items for systematic scoping review and meta-analysis (PRISMA) protocols.^13^

#### Search strategy

A systematic search of the literature was performed using PubMed via the official Application Programming Interface (API) of the National Library of Medicine (NLM) from inception until November 2021 to identify the quality-of-care indicators for diabetes, hypertension and CVDs, breast cancer, cervical cancer, chronic respiratory diseases, and childhood cancers. The constant term “Quality Indicators, Health Care” was used as the major MeSH term, combined with MeSH terms and keywords for each disease. The complete search strategy for each condition is provided in Supplementary Appendix 1: Supplementary Table S1.

The retrieved articles were imported into Zotero (Roy Rosenzweig Center for History and New Media, George Mason University in Fairfax County, Virginia, USA) and shared with the co-authors.

#### Study selection

**Fig. 1:**
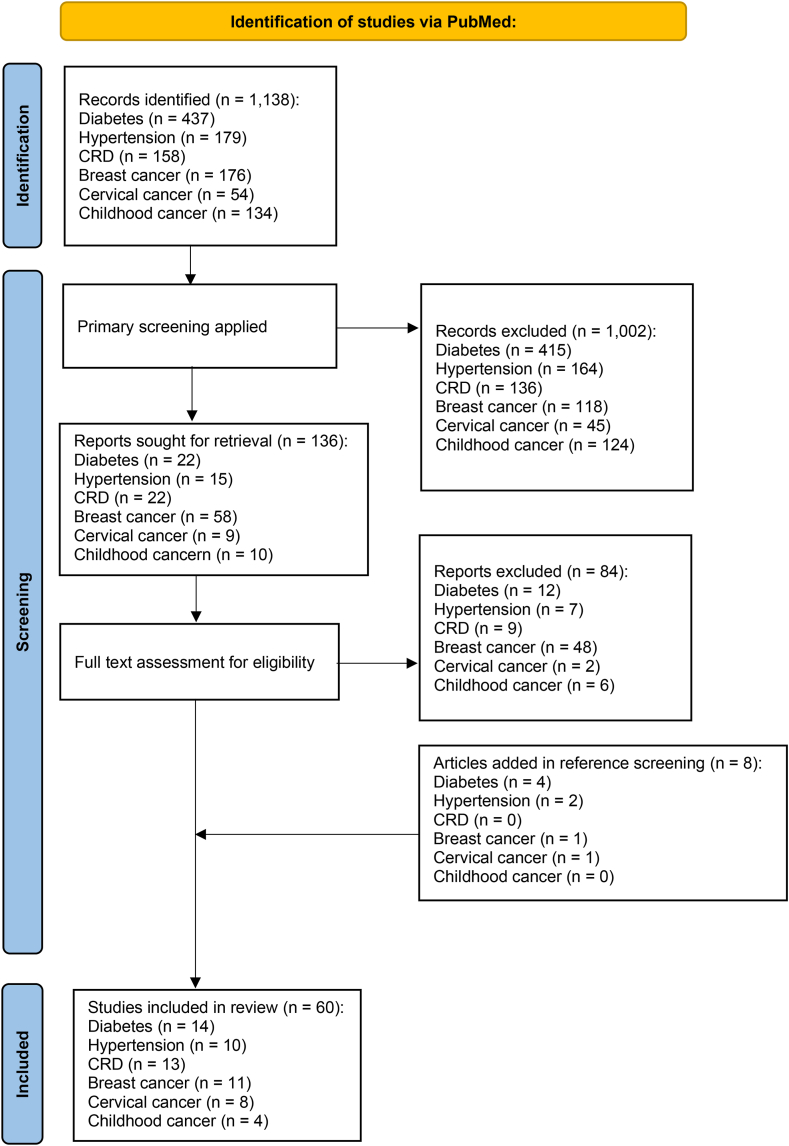
Flow chart of the final included studies in the review based on the PRISMA statement.

After removing the duplicates, due to the high number of articles, the retrieved articles were divided into three groups, and each group was reviewed by two independent researchers, a total of five researchers conducted this phase. The full texts of the selected articles were retrieved, and each article was reviewed by two independent researchers. In addition, manual screening was carried out for references of included articles to complement the literature body. Studies were included if they reported quality of care indicator for each of the diseases in primary care setting. In the case of discrepancies, a consensus was reached through discussion and the opinion of a third reviewer. In the initial phase, studies were included if they reported quality of care indicators for any of the diseases at a facility level and were excluded if the full text was unavailable, the language was not English, or they were not original. Study characteristics (author, design, and journal) and quality of care indicators were extracted (Fig. 1). A table consisting of the quality-of-care indicators was designed for each disease. We combined similar indicators without loss of content and listed them only once and provided the corresponding records. After the Delphi process and determination of the quality-of-care indicators, we removed the studies that provided indicators that the experts did not verify.

### Role of funding source

This study was funded internally by WHO. There were no payments to participants. WHO had a central role in the study design, coordination, data collection, and implementation. Data analysis and interpretation were conducted by the study authors in consultation with WHO technical teams. The writing of the report was led by the authors, and the views expressed are those of the authors and do not necessarily reflect the decisions or policies of WHO.

## Results

A draft set of 86 indicators was provided by the initial expert panel, 21 (1:4; 24%) of which were core. The full list of these indicators is presented in Supplementary Appendix 2. Disease-related indicators in addition to the cross-cutting ones were separately sent to a total of 104 participants of the expert panel based on their area of expertise.

### Delphi survey

Of a total of 104 invitations sent via electronic mail, 66 (63%) individuals completed the survey. After grouping experts based on their field of expertise, the number of respondents for each disease domain was 32 for hypertension and CVDs, 34 for diabetes mellitus, 19 for asthma and COPD, and 23 for cancer-related diseases including breast, cervical, childhood, and general cancers.

Of all respondents, 28 (42.4%) were female. Responses were received from participants in countries representing all WHO regions. Table 1 presents the demographic characteristics of participants in the Delphi process.

**Table 1:** Demographic characteristics of the Delphi panel.

Variables	Total	Hypertension & cardiovascular diseases	Diabetes mellitus	Asthma and chronic obstructive respiratory disease	Cancer-related diseases
**Sex**					
Female	28	10 (35.7%)	15 (53.6%)	12 (42.9%)	10 (35.7%)
Male	38	22 (57.9%)	19 (50.0%)	7 (18.4%)	13 (34.2%)
**WHO region**					
African	9	5 (55.6%)	5 (55.6%)	2 (22.2%)	4 (44.4%)
Americas	14	9 (64.3%)	7 (50.0%)	1 (7.1%)	4 (28.6%)
Eastern Mediterranean	7	3 (42.9%)	3 (42.9%)	3 (42.9%)	2 (28.6%)
European	12	4 (33.3%)	6 (50.0%)	3 (25.0%)	3 (25.0%)
South-East Asia	7	3 (42.9%)	3 (42.9%)	3 (42.9%)	2 (28.6%)
Western Pacific	5	2 (40.0%)	3 (60.0%)	1 (20.0%)	2 (40.0%)
Not specified	12	6 (50.0%)	7 (58.3%)	6 (50.0%)	6 (50.0%)

**Fig. 2:**
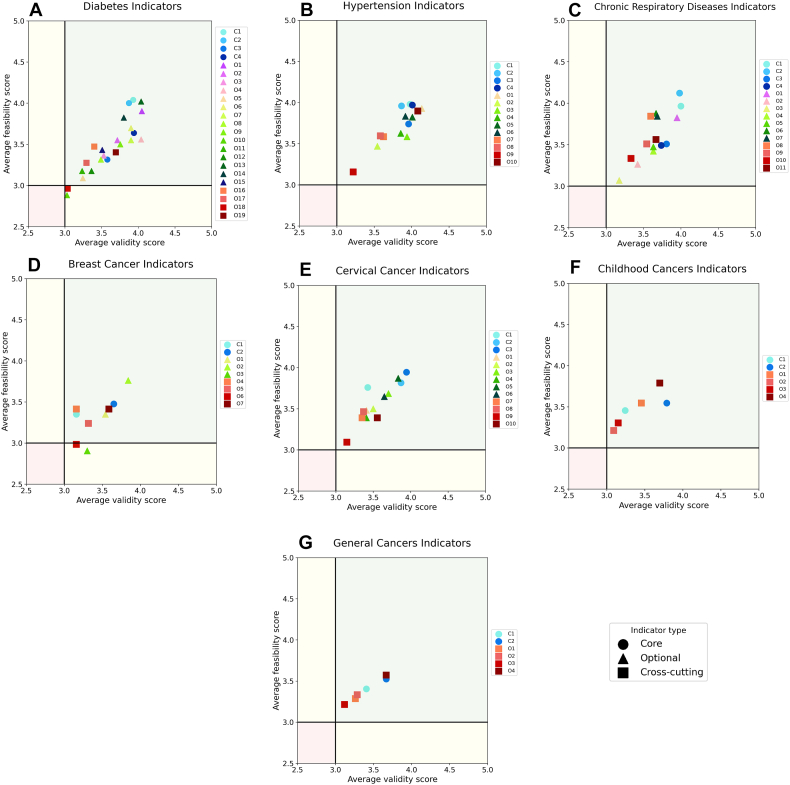
Average feasibility and validity scores of the 86 primary indicators during the Delphi process (Full names of the primary indicators are provided in the Supplementary Appendix 2). Panels: (A) Diabetes indicators; (B) Hypertension indicators; (C) Chronic respiratory diseases indicators; (D) Breast cancer indicators; (E) Cervical cancer indicators; (F) Childhood cancers indicators; (G) General cancers indicators.

Analysis of the survey responses pertaining to seven domains by relevant experts revealed that all core and optional indicators consistently demonstrated robust validity and feasibility, as reflected by their average scores surpassing the threshold of 3.0. However, there was variability in expert ratings for some indicators. As presented in Fig. 2, while the average validity scores of all indicators were higher than 3, the feasibility scores of two optional indicators were slightly below three; the score of “referral for lymphedema evaluation for patients following breast cancer treatment (O3)” indicator among breast cancer experts and the score of “severe hypoglycemia among people with diabetes (O10)” indicator among diabetes mellitus experts, which were discussed in the second round of focused expert panel meetings. During the qualitative assessment of the indicators, a total of 1070 comments were received, of which 14 resulted in major modifications. Through successive Delphi panel rounds and expert consultations, the initial set of 86 indicators underwent rigorous refinement. This process led to the removal of less feasible or redundant indicators, the consolidation of overlapping ones, and the inclusion of several newly prioritized indicators. The final set comprised 81 indicators: 53 core clinical/programmatic indicators and 28 cross-cutting system-level indicators categorized as optional (Table 2).

**Table 2:** Description of the qualitative Delphi survey.

Diseases	Number of respondents	Number of received comments
Hypertension and CVDs	32	246
Diabetes mellitus	34	320
Chronic respiratory diseases	19	187
Breast cancer	19	130
Cervical cancer	16	117
General cancers	13	36
Childhood cancers	10	34

### The final set of indicators

The final Delphi process yielded a set of 81 indicators, of which 22 were categorized as core indicators—those deemed essential for tracking NCD care in all healthcare settings. The remaining 59 were classified as optional indicators, which, while valuable, may be less feasible to collect in resource-limited settings. Core indicators included essential service delivery measures, such as the availability of hypertension medications and the proportion of diabetic patients achieving glycemic control. Optional indicators included more detailed measures of complications and secondary prevention efforts. The hypertension and CVDs set of indicators included four core indicators on assessing the availability of hypertension and CVDs core medicines, a functional blood pressure measuring device, the disease control among people with hypertension. There were four core indicators that mainly monitored diabetes mellitus services. These included three at inputs/process level assessing the availability of the core medicines and diagnostic tests; and one at outcome level assessing the glycemic control among patients with diabetes. The Delphi panel in asthma and COPD assessment group agreed to include four core indicators: two for inputs/processes assessment and two for outcome evaluation.

For breast cancer two core indicators were determined, both of which targeted the women aged 30–49 years with suspicious signs and/or symptoms. These indicators assess the clinical breast evaluation for early diagnosis and the timeliness of the referral for diagnosis. Cervical cancer’s Delphi panel defined four core indicators assessing the inputs and outputs of cervical cancer. For childhood cancer and general cancers, there were two core indicators for early diagnosis in each disease indicator set. All Delphi panel experts across the groups agreed on the inclusion of all four cross-cutting indicators as optional indicators for each disease. Table 3 presents the whole set of core and optional indicators for each disease group.

**Table 3:** The final set of indicators for primary care facility-based NCD monitoring system framework.

Input/process	Output	Outcome
**Hypertension and cardiovascular diseases**		
Availability of hypertension core medicines^a^ Availability of cardiovascular disease core medicines^a^ Availability of a functional blood pressure measuring device^a^	Assessment of cardiovascular disease risk (aged ≥ 40 years) among people aged over 40 years using WHO CVD risk charts. Screening for hypertension among people aged 18 and over adults as part of routine service. Hypertension detection from opportunistic screening Assessment for chronic kidney disease among people newly diagnosed with hypertension.	Blood pressure control among people with hypertension^a^ Blood pressure control among people with hypertension (follow-up)
**Diabetes mellitus**		
Availability of diabetes core medicines^a^ Availability of plasma glucose testing^a^ Availability of Hemoglobin A1c testing^a^	Pharmacological treatment among people with diabetes Statin therapy among people with diabetes Pharmacological treatment for chronic kidney disease among people with diabetes Pharmacological treatment for hypertension among people with diabetes Assessment for diabetic chronic kidney disease among people with diabetes Assessment for diabetic foot among people with diabetes Referral for retinopathy screening among people with diabetes	Glycaemic control among people with diabetes^a^ Glycaemic control among people with diabetes (follow-up) Chronic kidney disease among people with diabetes Lower-limb amputation among people with diabetes Blindness among people with diabetes
**Asthma and chronic obstructive pulmonary disease rational**		
Availability of asthma core medicines^a^ Availability of chronic obstructive pulmonary disease core medicines^a^ Availability of peak flow meter and mouthpiece	Asthma diagnosis using peak flow measurement Chronic obstructive pulmonary disease diagnosis using peak flow measurement Treatment among people with asthma Treatment among people with chronic obstructive pulmonary disease	Asthma control^a^ Chronic obstructive pulmonary disease control^a^ Emergency visit among people with asthma Emergency visit among people with chronic obstructive pulmonary disease
**Breast cancer**		
	Clinical breast evaluation for early diagnosis of breast cancer among women aged 30–49 years with signs and/or symptoms associated with breast cancer^a^ Timeliness of referral for breast cancer diagnosis among women aged 30–49 years with associated signs and/or symptoms of breast cancer who had suspicious findings from clinical breast evaluation^a^ Referral for mammography screening among women aged 50–69 years Timeliness of breast cancer confirmatory diagnosis among women aged 30–49 years with suspicious findings from clinical breast evaluation Timeliness of breast cancer treatment among women aged 30–49 years with suspicious findings from clinical breast evaluation	
**Cervical cancer**		
Availability of human papillomavirus testing^a^ Availability of Pap smear testing Availability of visual inspection with acetic acid testing	Cervical cancer screening with high performance test among women aged 30–49 years^a^ Cervical cancer screening among women aged 30–49 years^a^ Cervical cancer screening test positivity among women aged 30–49 years^a^ Cervical cancer rescreening among women aged 30–49 years Pre-invasive cervical disease treatment among women aged 30–49 years Timeliness of referral for cervical cancer diagnosis among women aged 30–49 years with suspicious findings from cervical cancer screening	
**Childhood cancers**		
	Clinical evaluation for early diagnosis of childhood cancer among children with signs and/or symptoms associated with cancer^a^ Timeliness of referral for childhood cancer diagnosis among children with associated signs and/or symptoms of childhood cancer who had suspicious findings from clinical evaluation^a^	
**General cancers**		
	Clinical evaluation for early diagnosis of cancer among people with signs and/or symptoms associated with cancer^a^ Timeliness of referral for cancer diagnosis among people with associated signs and/or symptoms of cancer who had suspicious findings from clinical evaluation^a^	

a Core indicators.

### Systematic scoping review

As presented in Fig. 1, A total of 1138 non-duplicated studies were identified from a comprehensive database search, focusing on quality indicators associated with hypertension and CVDs, diabetes, asthma and COPD, breast cancer, cervical cancer, childhood cancer, and cross-cutting indicators. Following an initial screening, 136 studies were considered for full-text retrieval. Subsequently, 84 studies were excluded during the full-text screening process, while eight additional studies were included after reference screening. Ultimately, 60 studies were included in the analysis, investigating quality-of-care indicators for the diseases (Supplementary Appendix 1: Supplementary Tables S2–S7). Among the studies included in the analysis, the Delphi methodology and retrospective studies were the most frequently employed, observed in 20 and nine studies, respectively. Conversely, population-based studies, pragmatic non-randomized controlled studies, practice guidelines, technical reports, time-series analysis, descriptive studies, and questionnaire-based surveys were each represented by only one study.

The frequency of indicator mentions varied among different disease categories in the systematic scoping review. Indicators pertaining to cancers were comparatively less commonly referenced compared to those related to hypertension and CVDs, diabetes, asthma and COPD. Among all the cancer indicators, only three indicators (timeliness of breast cancer treatment among women aged 30–49 years with suspicious findings from clinical breast evaluation, cervical cancer screening among women aged 30–49 years, cervical cancer screening test positivity among women aged 30–49 years) were mentioned more than three times in the literature. Conversely, among the indicators related to hypertension and CVDs, diabetes, asthma and COPD, five, eight and eight indicators, respectively, were mentioned more than three times. Considering the core indicators for hypertension and CVDs, diabetes, and asthma, and COPD, indicators associated with the availability of medicine and health equipment received less attention compared to indicators related to the evaluation of disease control in the population, as evidenced by the literature review. Additionally, among the optional indicators, cross-cutting indicators including completeness and timeliness of reporting by health facilities and facilities receiving supervisory visit were not mentioned in the literature regarding all diseases. Supporting evidence for each indicator are presented in the Supplementary Appendix 2.

## Discussion

Facility-based NCD monitoring systems can be pivotal in empowering public health authorities to make informed decisions through the routine collection, compilation, and analysis of patient and facility indicators. These systems serve as a critical tool for assessing the overall performance of healthcare facilities and the individuals working within them, ensuring that healthcare providers deliver quality care consistently. The primary objective of the facility-based NCD monitoring system is to monitor and evaluate service delivery in terms of both quality and utilization. Unlike facility surveys, which offer only a snapshot of performance at a particular moment, facility-based NCD monitoring systems provide an ongoing and dynamic view of the healthcare delivery process, enabling continuous monitoring and improvement. Until now, despite the advancements in health management information systems, a crucial gap existed in the standardization and comparability of the indicators collected from diverse sources and at various times. The present study has marked a significant milestone by introducing standardized indicators with unified definitions, clear purposes, and consistent calculation methods. This standardized framework ensures that indicators remain comparable across different healthcare facilities, subnational regions, national levels, and even on the global stage. It facilitates not only cross-sectional assessments but also the ability to track progress and trends over time, which is essential for effective healthcare management and policy development. Moreover, the efforts outlined in this study have the potential to serve as a valuable model for countries where regular data collection within their NCD healthcare systems has historically been lacking. By demonstrating the benefits of the facility-based NCD monitoring system in enhancing healthcare delivery and monitoring, this initiative can inspire and guide other member states in establishing similar systems to strengthen their healthcare infrastructure and improve patient outcomes.^14^

NCDs surveillance is a system composed of interconnected pieces that contribute to our understanding and management of a set of conditions with a high societal and health service burden. The first piece involves the vital registries including death and cancer registration systems, which provide the foundational framework to decipher the intricate patterns of mortality related to NCDs incidence. This information is used to evaluate the impact of NCDs on both individuals and populations. The second piece takes the form of policy and governance tracking surveys, exemplified by NCD country capacity surveys. This piece acts as the scaffold that steadfastly holds the puzzle together. It assesses a nation’s capacity to tackle the challenges posed by NCDs, akin to evaluating the structural support beams and health system determinants. Through these surveys, healthcare authorities scrutinize policies, resources, and infrastructure, which collectively underpin our ability to combat NCDs effectively. The third piece takes the form of population-based studies, and in this realm, WHO STEPwise approach to NCD risk factor surveillance (STEPS), Global School-based Student Health Survey (GSHS), Global Youth Tobacco Survey (GYTS), and Global Adult Tobacco Survey (GATS), and other similar studies emerge as a luminary. These surveys allow us to craft a meticulous canvas of the broader epidemiological landscape of NCDs, skillfully illuminating risk factors and trends within a nation’s population. They provide the necessary context for crafting effective public health strategies. Lastly, the fourth piece is the facility-based NCD monitoring system, serving as the bridge that connects the broader landscape to the intricate details of NCD management. Facility-based NCD monitoring system offers valuable insights into the quality of care provided, the performance of healthcare providers, and the dynamics within healthcare facilities. It allows us to examine a wide range of impacts and interventions related to NCDs, encompassing not only the prevalence of NCDs but also the patient’s journey and the effectiveness of healthcare services in real-time. The true power of a comprehensive NCD surveillance system lies in how these pieces interact harmoniously. Vital registries illuminate the ultimate outcomes, policy and governance tracking surveys scrutinize the structural framework, population health surveys provide the grand panorama, and the facility-based NCD monitoring system offers ground-level, real-world data. Collectively, these pieces form a multidimensional approach that paints a holistic and actionable picture of NCDs. This collective insight becomes the guiding compass for shaping effective healthcare policies and interventions, addressing not only the prevalence of NCDs but also ensuring quality care and improved health outcomes for individuals and populations.

The development of this NCD surveillance system marks a significant achievement in the field of public health data collection. The methodology employed in creating this guidance holds promise as a valuable blueprint for extending its scope to encompass additional NCD diseases. By incorporating new modules into the existing guidance, we can effectively expand its applicability, addressing a broader spectrum of NCDs and thereby enhancing our capacity to tackle these conditions comprehensively. One of its notable accomplishments lies in its ability to unify comparisons across various levels of the healthcare system. The framework of this guidance aligns well with the primary healthcare (PHC) framework, facilitating seamless data integration at the PHC level. This synergy between the guidance and PHC principles promotes the optimization of healthcare resources and the delivery of patient-centered care, ultimately contributing to improved NCD management and outcomes. This unification ensures that data can be seamlessly analyzed and compared, whether at the facility, subnational, national, or global level. Such consistency is invaluable for shaping effective healthcare policies and interventions.

The facility-based NCD monitoring systems could address the information needs of countries in establishing a routine health information system for NCDs. They could cater to different levels of data collection, from triage to physician’s records, patient files, and reporting forms to higher level. They could also accommodate various data collection methods, including paper and digital forms. Furthermore, facility-based NCD monitoring systems could offer aggregate digital platforms, making data readily available online, a significant advancement from paper-based systems where only aggregated data were accessible online. Additionally, the guidance offers the potential to foster enhanced data linkage between primary, secondary, and tertiary healthcare levels. Establishing robust data connections between these levels is a significant advantage, as it promotes a comprehensive understanding of the NCD landscape across the healthcare continuum. This interconnectedness not only enables a more holistic assessment of patient journeys and outcomes but also aids in the development of effective strategies for NCD prevention and management as well as referral systems.

The guidance provides a standardized framework for indicator definition and data collection, ensuring comparability and uniformity of health data both nationally and internationally. This standardization enables policymakers to benchmark performance, identify best practices, and promote evidence-based healthcare decisions. Additionally, the modular nature of the guidance allows countries flexibility in adopting indicators based on resource availability, infrastructure readiness, and specific healthcare priorities. The proposed integration with digital platforms further creates an opportunity to enhance data accuracy, reduce reporting burden, and facilitate real-time data-driven decision-making.

Despite these opportunities, implementing this guidance presents several challenges. Financial constraints, particularly in low- and middle-income countries, could significantly limit sustainable investment in monitoring infrastructures and digital solutions. Additionally, concerns related to data privacy, confidentiality, and security could hinder the adoption of digital tools without clear legal frameworks and robust cybersecurity measures. The human resource challenge also remains substantial, as adequately trained personnel capable of managing complex data systems are scarce in many settings. To overcome these hurdles, tailored support mechanisms—including training programs, sustainable funding streams, and robust regulatory frameworks—will be essential. The success of this guidance thus depends on countries’ abilities to address these implementation barriers strategically and collaboratively.

Facility-based NCD monitoring system distinguishes between core and optional indicators, acknowledging the limitations in human resources and high workloads. By offering a handful of core indicators, it simplifies the data collection process and recognizes the need for efficiency. The adaptability and dynamic nature of the system also allow for expansion to include other diseases, as exemplified by the impending inclusion of dental health in the guidance. Furthermore, the meta-data design allows countries to make specific adjustments without compromising data comparability. This adaptability ensures that the system remains relevant and responsive to evolving technological advancements and the progress of individual countries. Periodic updates and revisions, scheduled every 3–5 years, will guarantee that the indicators remain in compliance with technological advances and countries’ development.

The advancement of electronic health records and the accumulation of various health datasets present tremendous opportunities for NCD surveillance, particularly when combined with flexible digital platforms that support data collection and management.^15^ While paper-based tools could be employed as accessible means of data collection for countries with very limited resources and also for less prevalent diseases, middle income and high income countries and high-volume and periodically collected data for high prevalence and high burden diseases, either on individual or aggregated level, would better be fed into a flexible digital platform which can be customized to support the collection and management of a wide range of health data, including data on NCDs.^16^ Electronic data collection could potentially be a feasible choice especially in settings with limited resources, manpower shortage, or high workload.^17^ Such means of data collection can contribute to more accurate data collection, improved analysis, and effective interventions.

Nevertheless, it’s crucial to acknowledge the system’s limitations. Currently, it primarily focuses on primary care settings, and plans are underway to incorporate facility-based NCD monitoring system modules for secondary and tertiary healthcare settings, recognizing the importance of comprehensive data collection. One significant omission of the facility-based NCD monitoring system is the absence of the patient’s perspective, as the system does not include patient-reported outcome measures. This limitation is slated for rectification, as a module dedicated to patient-reported outcomes is in the pipeline. Additionally, finance data, a critical component of healthcare management, is currently not included in the system. This is a pertinent issue, especially in settings where primary care is predominantly carried out by the public sector. The lack of easily calculable service costs hinders comprehensive financial analysis. Plans are being explored to address this gap, provided that better data can be obtained and recorded.

To effectively implement this NCD surveillance system, several key requirements must be met. Infrastructure development is paramount, including capacity building, particularly in terms of human resources. Installation of data collection platforms is crucial, considering the diverse preferences among regions for paper-based or digital packages. These platforms need to meet specific requirements such as data privacy, end-to-end encryption, and offline functionality. The data collection platforms need to be evaluated by users to explore their compatibility with the flow of patients and data as well as user acceptability using End User Testing. It also needs to include tools for analysis, visualization, and interpretation of health data, which can help identify trends, prioritize interventions, and monitor progress toward health goals. Also, action plan developer tools which provide granular information and step-by-step instructions on service delivery performance to achieve national and global service delivery targets are crucial. Moreover, the platform should allow for the sharing of health data across different levels of the health system and with external partners, which can facilitate collaboration and coordination. In this sense, WHO would support all software solutions meeting the abovementioned criteria, whose source code is freely available and could be modified and improved by anyone. Such valuable tools could support countries’ efforts to collect standardized health indicators for NCDs and produce a sustainable source of data for making informed decisions at all levels of the health system. The use of a standardized set of indicators would also facilitate comparisons between countries and regions, which could help identify best practices and areas for improvement.^18^

To ensure data quality, robust data quality review systems should be established, and interoperability with existing health information systems, like DHIS-2 or digital adaptation kits (DAK) developed by WHO, should be considered for smoother integration.

As the implementation of standardized indicators for NCD monitoring progresses, it is essential to acknowledge the challenges. One major hurdle is the lack of uniformity in NCD-oriented programs across countries, resulting in discrepancies in data collection and reporting. Varying infrastructures, IT capabilities, and donor support further contribute to the inconsistency of NCD surveillance efforts. Moreover, inadequate training and capacity-building initiatives among healthcare professionals hinder the proper implementation and utilization of standardized indicators. Addressing this challenge via proper trainings is crucial to ensure maximum participation, accurate data collection, and reporting at all levels. Overburdened healthcare workers often struggle to balance patient care with data collection responsibilities. The high patient volume, especially in low-resource settings, can lead to data collection gaps and potential inaccuracies. The effective utilization of NCD data for program management and decision-making is an ongoing challenge. Developing robust service management systems that integrate data analysis and interpretation tools is essential for leveraging the collected information to drive actionable interventions. Maintaining data accuracy, completeness, and reliability is crucial for sound NCD surveillance and new technologies such as artificial intelligence might be future perspectives to reduce the burden of data recording and patient management in primary health care delivery settings. Ensuring data quality requires ongoing monitoring, validation, and feedback mechanisms to minimize errors and improve the overall reliability of collected data. Efforts to address NCD monitoring have been fragmented, with multiple global initiatives and digital platforms developed by NGOs and the private sector. Establishing stronger public-private partnerships and harmonizing efforts are critical to streamline NCD surveillance activities and avoid duplication.^19^^,^^20^ To enhance NCD monitoring, the development of additional modules regarding other aspects of health besides primary healthcare within existing platforms should be prioritized. Addressing the specific needs of secondary and tertiary-level facilities and integrating patient-reported outcome measures will further enrich the platform’s capabilities. Moreover, integrating patient-reported outcome measures into NCD surveillance can provide valuable insights into the patient’s experience with healthcare services. Leveraging popular messaging mobile applications and adopting a patient-centered approach to quality of care aligns with international standards and recommendations. Future directions should focus on the development of tools that facilitate target setting and action planning. These tools will aid countries in formulating effective strategies to address NCDs based on standardized indicators and support the achievement of global targets.

This study had many limitations. The study employed a Delphi method, which, although robust, relies heavily on expert consensus. Consequently, the quality of the identified indicators may be influenced by the expertise, background, and subjective judgment of the participating panel members. Different expert panels might have yielded different sets of indicators, potentially affecting generalizability. Furthermore, despite efforts to gather diverse perspectives, non-response bias might have influenced the results, given that not all invited experts participated fully. In addition, the systematic scoping review conducted in this study was limited to English-language publications, possibly omitting valuable insights from literature published in other languages. This language restriction may have introduced publication bias, affecting the comprehensiveness of the identified indicators. The variability in the reporting of quality indicators across existing literature also posed a limitation, making direct comparisons challenging and potentially limiting the generalizability of findings. Moreover, the proposed monitoring system currently lacks indicators capturing patient-reported outcomes and economic (cost-related) data, representing significant gaps that may limit the holistic evaluation of healthcare quality and economic sustainability. Lastly, practical limitations related to the implementation of the monitoring system—such as financial constraints, workforce shortages, limited technological infrastructure, and data privacy and security concerns—were identified but will require specific attention in future implementation efforts.

In this study, we outlined the process of developing facility-based NCD indicators and conducted a systematic scoping review to assess their usage in the scientific literature. Facility-based NCD monitoring system ensures the consistent provision of quality care and helps countries monitor progress toward their NCD-related goals. The guidance introduced here not only standardizes key NCD indicators but also offers a blueprint for extending its scope to cover more NCDs. Its alignment with the primary healthcare framework enhances data integration, optimizing healthcare resources and fostering patient-centered care. To tackle challenges such as data standardization and capacity building, it is essential to build strong partnerships between the public and private sectors and coordinate their efforts. The future of NCD monitoring should focus on expanding the platform’s capabilities, integrating patient perspectives, and developing tools for better target setting and action planning. In essence, this study lays the groundwork for improved NCD surveillance and healthcare management, ultimately striving for better health outcomes worldwide.

## Contributors

Conceptualization: FF, AQ, LR, SS, BA, PB, EF, BH, AI, TK, RO, GR, SR, FR; Data Curation: AQ, MRM, AG, MHF, SHG, MAK; Formal Analysis: AG, MHF, MRM, MAK, SHG; Investigation: AQ, SHG, MRM, AG, MHF, MAK; Methodology: FF, AQ, LR, SS, BA, PB, EF, BH, AI, TK, RO, GR, SR, FR; Project Administration: FF, AQ, LR, SS, BA, PB, EF, BH, AI, TK, RO, GR, SR, FR; Resources: FF; Supervision: FF; Validation: FF, AQ; Visualization: AG, MHF, MRM, MAK, SHG; Writing – Original Draft: SHG, AQ, MRM, AG, MHF, MAK; Writing – Review & Editing: FF, AQ, LR, SS, BOA, PB, EF, BH, AI, TK, RO, GR, SR, FR, MRM, AG, MHF, SHG, MAK, KA, AA, OA, CA, HAC, RMA, CAn, ZA, SB, AB, PBs, SBe, KB, JB, NC, RC, NCa, CC, SRC, MAC, AC, ND, SD, GD, JMD, WDG, CBD, ID, RED, GD, BBD, UE, HEB, AES, ME, JF, HF, PF, SFA, AGa, EG, RG, SG, WJ, EJ, PJ, RK, SK, TL, BL, MLL, TLT, NL, SL, MM, BM, YM, AM, AMo, SM, RM, MMu, RN, MN, PO, DO, GO, DOnd, POr, MOSM, MO, KR, IR, JFR, AR, JR, RS, VS, JT, MT, ET, TT, CV, LV, KV, HX, CHY. All authors read and approved the final version of the manuscript. AQ, FF, SHG, MRM, MAK, AG and MHF have verified the underlying data.

## Data sharing statement

All metadata for the developed indicators are available in Supplementary Appendix 2. Additional materials, including summary results from the Delphi process and extracted data from the systematic scoping review, are available upon reasonable request to the corresponding author. No individual-level or patient-identifiable data were collected or used in this study.

## Declaration of interests

Dr. Evelyn Jiagge has received institutional payments from Pfizer and Genentech via Henry Ford Health. Kazem Rahimi reports grants from the National Institute for Health Research (NIHR304997), Medical Research Council (MR/Y030419/1), British Heart Foundation (FS/PhD/22/29321, FS/PhD/21/29110, FS/PhD/25/29632), European Union (101080430), Roche (R94776/CN002), and Novo Nordisk Oxford Big Data Partnership. He receives personal royalties from Lucem Health and honoraria as Editor-in-Chief of Heart and for speaking engagements with Radcliffe Cardiology. He also serves on Medtronic’s Renal Denervation Advisory Board.

Norm RC Campbell holds unpaid advisory roles with the World Hypertension League, HEARTS in the Americas (PAHO/WHO), and as Vice Chair of the Canadian Hypertension Coalition. Patrick J. O’Connor has received institutional grant support from the U.S. National Institutes of Health for research unrelated to this work. Sumit Gupta has received consulting fees and honoraria from Amgen for advisory work and lectures on the role of immunotherapy in acute lymphoblastic leukemia (ALL).

Abdul Basit, Jean-Marie Dangou, Hicahm El Berri, Asma El Sony, Paola Friedrich, Soad Fuentes-Alabi, Ratnasabathipillai Kesavan, Bagher Larijani, Mauricio Maza, Gojka Roglic, Rengaswamy Sankaranarayanan, and Josaia Tiko were required to sign the Confidentiality Undertaking and Declaration of Interest forms via the WHO platform, which did not allow experts with any potential competing interests to participate in the panel meeting.
