# Supplementary material for: World health Organization’s guidance for tracking non-communicable diseases towards sustainable development goals 3.4: an initiative for facility-based monitoring
Source: eClinicalMedicine. 2025 Jul 2;85:103304. doi: 10.1016/j.eclinm.2025.103304 (PMC12269858; doi:10.1016/j.eclinm.2025.103304)
Supplement: Supplementary Appendix 1 [file mmc1.docx]

**Supplementary appendix 1**

Supplement to: World Health Organization's Guidance for Tracking Noncommunicable Diseases towards Sustainable Development Goals 3.4: An Initiative for Facility-Based Monitoring

**Supplementary Tables**

| **Supplementary** **Table 1**. Search queries used in the systematic review | |
| --- | --- |
| **Disease** | **Search Query** |
| Diabetes | "Diabetes Mellitus"[Mesh] OR "Diabetes Mellitus"[Title/Abstract] |
| Hypertension | "Hypertension"[Mesh] OR "Hypertension"[Title/Abstract] |
| Chronic Respiratory Diseases | "Pulmonary Disease, Chronic Obstructive"[Mesh] OR "Asthma"[Mesh] OR "Lung Diseases, Obstructive"[Mesh] OR "Asthma"[Title/Abstract] |
| Breast Cancer | "Breast Neoplasms"[Mesh] OR "Breast Cancer"[Title/Abstract] |
| Cervical Cancer | "Uterine Cervical Neoplasms"[Mesh] OR "Cervical Cancer"[Title/Abstract] |
| Childhood Cancers | ("neoplasms"[MeSH] OR cancer[Title/Abstract] OR neoplas*[Title/Abstract] OR carcinoma[Title/Abstract] OR malignan*[Title/Abstract] OR malignancy[Title/Abstract] OR tumor*[Title/Abstract] OR tumour*[Title/Abstract] OR oncolog*[Title/Abstract] OR metasta*[Title/Abstract] OR leukemia*[Title/Abstract] OR lymphoma*[Title/Abstract] OR "brain cancer" [Title/Abstract] OR (brain[Title/Abstract] AND (cancer*[Title/Abstract] OR neoplasm*[Title/Abstract])) OR brain tumor*[Title/Abstract] OR brain tumour*[Title/Abstract] AND ("Child"[Mesh] OR "Adolescent"[Mesh] OR "Pediatrics"[Mesh] OR "Infant" [Mesh] OR children[Title/Abstract] OR childhood[Title/Abstract] OR kids[Title/abstract] OR youth[Title/Abstract] OR juvenile[Title/Abstract] OR Infant*[Title/Abstract] OR adolescen*[Title/Abstract] OR adolescent[Title/Abstract] OR paediatric[Title/Abstract] OR pediatric[Title/Abstract] OR teen*[Title/Abstract] OR teenager*[Title/Abstract]) |

| **Supplementary Table 2**. Titles retrieved for hypertension and CVDs core and optional primary care facility-based NCD monitoring system framework indicators | | | | |
| --- | --- | --- | --- | --- |
| **Title** | **Type** | **Journal** | **Impact Factor** | **Corresponding author** |
| Quality indicators for general practice: which ones can general practitioners and health authority managers agree are important and how useful are they? | Delphi | Journal of public health medicine | - | Stephen Campbell |
| 2019 AHA/ACC Clinical Performance and Quality Measures for Adults with High Blood Pressure: A Report of the American College of Cardiology/American Heart Association Task Force on Performance Measures | Practice Guideline | American College of Cardiology | 24·1 | Donald E Casey Jr |
| A new approach for measuring quality of care for women with hypertension | Delphi | JAMA Internal Medicine | 21·9 | Steven Asch |
| Quality indicators for the prevention and management of cardiovascular disease in primary care in nine European countries | Delphi | European Journal of Preventive Cardiology | 7·8 | Stephen Campbell |
| Clinical governance in primary care groups: the feasibility of deriving evidence-based performance indicators | Retrospective | BMJ Quality & Safety | 7 | Alastair McColl |
| Quality indicators for cardiovascular primary care | Delphi | The Canadian journal of cardiology | 5·2 | Frederick Burge |
| The Quality of Primary Care in a Country with Universal Health Care Coverage | Retrospective | Journal of General Internal Medicine | 5·1 | Nicolas Rodondi |
| Does quality of care for hypertension in primary care vary with postcode area deprivation? An observational study | Observational Study | BMC Health Services Research | 2·5 | Nicholas Steel |
| Quality of care for hypertension in the United States | Comparative Study | BMC Cardiovascular Disorders | 2·3 | Steven Asch |
| Developing a framework of, and quality indicators for, general practice management in Europe | Delphi | Family Practice | 1·7 | Yvonne Engels |

| **Supplementary Table 3**. Titles retrieved for diabetes mellitus core and optional primary care facility-based NCD monitoring system framework indicators | | | | |
| --- | --- | --- | --- | --- |
| **Title** | **Type** | **Journal** | **Impact Factor** | **Corresponding author** |
| Improving Health Outcomes of People with Diabetes Mellitus: Target Setting to Reduce the Global Burden of Diabetes Mellitus by 2030 | Technical document | N/A | N/A | Noncommunicable diseases |
| Quality of Care of the Initial Patient Cohort of the Diabetes Collaborative Registry | Cross-sectional | Journal of the American Heart Association | 4·605 | Suzanne V. Arnold |
| Quality performance and associated factors in Swiss diabetes care - A cross-sectional study | Randomized Controlled Trial | PLoS One | 3·24 | Rahel Meier |
| Selecting indicators for the quality of diabetes care at the health systems level in OECD countries | Delphi | International Journal for Quality in Health Care | 2·038 | Antonio Nicolucci |
| Measuring adherence rate to quality indicators for diabetes care identified by primary health care in Bahrain | Cross-sectional | Saudi Medical Journal | 1·484 | Basem A Al-Ubaidi |
| Optimal type 2 diabetes mellitus management: the randomised controlled OPTIMISE benchmarking study: baseline results from six European countries | Randomized Controlled Trial | European Journal of Preventive Cardiology | 7.804 | Michel P Hermans |
| Developing a set of indicators to monitor quality in ambulatory diabetes care using a modified Delphi panel process | Delphi | International Journal for Quality in Health Care | 2·038 | Geetha Mukerji |
| Swiss quality and outcomes framework: quality indicators for diabetes management in Swiss primary care based on electronic medical records | Cross-sectional | Gerontology | 5·14 | Sima Djalali |
| Quality indicators for ambulatory care for older adults with diabetes and comorbid conditions: A Delphi study | Delphi | PLoS One | 3·24 | Walter P. Wodchis |
| Development of quality indicators for type 2 diabetes, extractable from the electronic health record of the general physician. A rand-modified Delphi method | Delphi | Primary Care Diabetes | 2·459 | Steve A.Van den Bulck |
| Prescribing quality indicators of type 2 diabetes mellitus ambulatory care | Delphi | BMJ Quality & Safety | 7·035 | Nikolay L Martirosyan |
| Quality indicators for type-2 diabetes care in practice guidelines: an example from six European countries | Review | Primary Care Diabetes | 2·459 | Johan Wens |
| Health literacy and quality of care of patients with diabetes: A cross-sectional analysis | Cross-sectional | Primary Care Diabetes | 2·459 | Emilie Zuercher |
| Incidence, prevalence, costs, and quality of care of type 1 diabetes in Italy, age 0-29 years: The population-based CINECA-SID ARNO Observatory, 2002-2012 | Cohort | Nutrition, Metabolism and Cardiovascular Diseases | 4·222 | Graziella Bruno |

| **Supplementary Table 4.**  Titles retrieved for chronic respiratory diseases core and optional facility-based patient and program monitoring indicators | | | | |
| --- | --- | --- | --- | --- |
| Title | Type | Journal | Impact Factor | Corresponding author |
| Impact of a patient-specific co-designed COPD care scorecard on COPD care quality: a quasi-experimental study | pragmatic non-randomised controlled study | NPJ primary care respiratory medicine | 3·231 | C Michael Roberts |
| Influence of government-driven quality assessment program on patients with chronic obstructive pulmonary disease | Comparative study | *Respiratory Research* | 5·631 | Jae-Hyun Lee |
| How robust are health plan quality indicators to data loss? A Monte Carlo simulation study of pediatric asthma treatment | Retrospective | Health services research | 3·402 | Bruce Stuart |
| Indicators for childhood asthma in Spain, using the Rand method | RAND method | Allergologia et immunopathologia | 1·667 | J. Ruiz-Canela-Cáceres |
| Is it time for a patient-centered quality measure of asthma control? | Review | The Journal of Allergy and Clinical Immunology: In Practice | 8·861 | Elizabeth Herman |
| Measuring the effects of health information technology on quality of care: a novel set of proposed metrics for electronic quality reporting | Delphi | The Joint Commission Journal on Quality and Patient Safety | 1·65 | Rainu Kaushal |
| Predictors of the quality of care for asthma in general practice: an observational study | Observational study | Family practice | 2·267 | Asmaa S Abdelhamid |
| Quality of asthma care under different primary care models in Canada: a population-based study | population-based study | BMC family practice | 2·398 | Teresa To |
| Comparative evaluation of two asthma care quality measures among Medicaid beneficiaries | Comparative study | Chest | 9·657 | Mihail Samnaliev |
| Quality of care for veterans with chronic diseases: performance on quality indicators, medication use and adherence, and health care utilization | cross-sectional | Population health management | 2·459 | Priest, Julie L |
| Reliability of medical group and physician performance measurement in the primary care setting | cohort | Medical care | 3·241 | Thomas Sequist |
| Withdrawing performance indicators: retrospective analysis of general practice performance under UK Quality and Outcomes Framework | Retrospective | The BMJ | 39·89 | Evangelos Kontopantelis |
| Evidence-based performance indicators of primary care for asthma: a modified RAND Appropriateness Method | RAND method | International Journal for Quality in Health Care | 2·038 | Teresa To |

| **Supplementary Table 5**. Titles retrieved for breast cancer core and optional facility-based patient and program monitoring indicators | | | | |
| --- | --- | --- | --- | --- |
| **Title** | **Type** | **Journal** | **Impact Factor** | **Corresponding author** |
| Breast cancer screening in the Czech Republic: time trends in performance indicators during the first seven years of the organised programme | Retrospective | BMC public health | 4·135 | Ladislav Dusek |
| Identifying Coordination and Continuity of Care Indicators for Population-Based Cancer Screening Programs: A Delphi Study. | Delphi | Nursing research | 2·364 | Montse García |
| Population-based Cancer Screening: Measurement of Coordination and Continuity of Care | Delphi | Nursing research | 2·364 | Montse García |
| Monitoring and evaluation of breast cancer screening programmes: selecting candidate performance indicators | Delphi | BMC cancer | 4·638 | Nadya Dimitrova |
| Improvement of Breast Cancer Patient Pathway Using EUSOMA Standards and European Guidelines. | comparative | Chirurgia | 0·77 | Nuh Zafer Cantürk |
| Variation in performance in low-volume mammography screening programmes: experience from Switzerland | Retrospective | Cancer epidemiology | 2·89 | J-L. Bulliard |
| A community breast center report card determined by participation in the national quality measures for breast centers program | Comparative | Breast | 4·254 | Jeffrey Landercasper |
| Quality indicators in breast cancer care: An update from the EUSOMA working group | review | European journal of cancer | 10·002 | Laura Biganzoli |
| Quality indicators in breast cancer care | Delphi | European journal of cancer | 10·002 | MR Del Turco |
| Effect of hospital volume on processes of care and 5-year survival after breast cancer: a population-based study on 25000 women | Retrospective | Breast | 4·254 | France Vrijens |
| Developing and measuring a set of process and outcome indicators for breast cancer | Delphi | Breast | 4·254 | Sabine Stordeur |

| **Supplementary Table 6**. Titles retrieved for cervical cancer core and optional facility-based patient and program monitoring indicators | | | | |
| --- | --- | --- | --- | --- |
| **Title** | **Type** | **Journal** | **Impact Factor** | **Corresponding author** |
| Performance indicators of organized cervical screening in Romagna (Italy) | comparative study | European journal of cancer prevention | 2·497 | L Bucchi |
| Extension of organised cervical cancer screening programmes in Italy and their process indicators | Retrospective | Epidemiologia e prevenzione | 1·901 | GUGLIELMO RONCO |
| Evaluating a quality improvement program for cervical cancer screening at an urban safety net clinic | comparative study | Health promotion practice | 1·111 | Robin L.Hills |
| Performance indicators collected from primary health centres included in organised cervical cancer screening programme in the Republic of Serbia | Retrospective | J BUON | 2·533 | Tamara Naumovic |
| The quality of Pap smears from the Brazilian cervical cancer screening program according to the Human Development Index | Time-series analysis | Cancer Prevention Research | 3·473 | Ricardo Filipe Alves Costa |
| Quality indicators of cervical cytopathology tests in the public service in Minas Gerais, Brazil | Descriptive Study | Revista Brasileira de Ginecologia e Obstetrícia | 0·92 | Alessandra Hermógenes Gomes Tobias |
| Impact of implementing 100% rapid review as a quality control tool in cervical cytology | cross-sectional Study | Acta cytologica | 2·319 | Juliana Magalhães |
| Cervical cancer screening policies and coverage in Europe | questionnaire-based survey | European Journal of Cancer | 9·162 | Ahti Anttila |

| **Supplementary Table 7**. Titles retrieved for childhood cancers core and optional facility-based patient and program monitoring indicators | | | | |
| --- | --- | --- | --- | --- |
| Title | Type | Journal | Impact Factor | Corresponding author |
| Measuring the Quality of a Childhood Cancer Care Delivery System: Assessing Stakeholder Agreement | Delphi | Value in Health | 5·725 | Mark L. Greenberg |
| Development of Quality Metrics to Evaluate Pediatric Hematologic Oncology Care in the Outpatient Setting | Delphi | Journal of Pediatric Hematology/Oncology | 1·289 | Sumit Gupta |
| Development of System Performance Indicators for Adolescent and Young Adult Cancer Care and Control in Canada | Delphi | Value in Health | 5·725 | Ronald D. Barr |
| Quality and capacity indicators for hospitalized pediatric oncology patients with critical illness: A modified delphi consensus | Delphi | Cancer Medicine | 4·452 | Anita V. Arias |
