# Supplementary material for: World health Organization’s guidance for tracking non-communicable diseases towards sustainable development goals 3.4: an initiative for facility-based monitoring
Source: eClinicalMedicine. 2025 Jul 2;85:103304. doi: 10.1016/j.eclinm.2025.103304 (PMC12269858; doi:10.1016/j.eclinm.2025.103304)
Supplement: Supplementary Appendix 2 [file mmc2.docx]

**Supplementary appendix 2**

Supplement to: World Health Organization's Guidance for Tracking Noncommunicable Diseases towards Sustainable Development Goals 3.4: An Initiative for Facility-Based Monitoring

Contents

[**The Primary set of indicators for primary care facility-based NCD monitoring system framework** 3](#_Toc184908321)

[**Hypertension indicators:** 3](#_Toc184908322)

[**Diabetes indicators** 4](#_Toc184908323)

[**Chronic respiratory diseases indicators** 6](#_Toc184908324)

[**Breast cancer indicators** 7](#_Toc184908325)

[**Cervical cancer indicators** 8](#_Toc184908326)

[**Childhood cancers indicators** 9](#_Toc184908327)

[**General cancers indicators** 10](#_Toc184908328)

[**Supporting evidence** 11](#_Toc184908329)

[**Supporting evidence for Hypertension and cardiovascular diseases indicators** 11](#_Toc184908330)

[*Input/Process indicators* 11](#_Toc184908331)

[*Output indicators* 11](#_Toc184908332)

[*Outcome indicators* 13](#_Toc184908333)

[**Supporting evidence for diabetes mellitus indicators** 15](#_Toc184908334)

[*Input/Process indicators* 15](#_Toc184908335)

[*Output indicators* 15](#_Toc184908336)

[*Outcome indicators* 20](#_Toc184908337)

[**Supporting evidence for asthma and chronic obstructive pulmonary disease indicators** 23](#_Toc184908338)

[*Input/Process indicators* 23](#_Toc184908339)

[*Output indicators* 23](#_Toc184908340)

[*Outcome indicators* 24](#_Toc184908341)

[**Supporting evidence for breast cancer indicators** 26](#_Toc184908342)

[*Output indicators* 26](#_Toc184908343)

[**Supporting evidence for cervical cancer indicators** 28](#_Toc184908344)

[*Input/Process indicators* 28](#_Toc184908345)

[*Output indicators* 28](#_Toc184908346)

[**Supporting evidence for childhood cancer indicators** 30](#_Toc184908347)

[*Output indicators* 30](#_Toc184908348)

[**Supporting evidence for general cancer indicators** 31](#_Toc184908349)

[*Output indicators* 31](#_Toc184908350)

[**Supporting evidence for cross-cutting indicators** 32](#_Toc184908351)

[*Input/Process indicators* 32](#_Toc184908352)

[*Output indicators* 32](#_Toc184908353)

[**References** 34](#_Toc184908354)

**The Primary set of indicators for primary care facility-based NCD monitoring system framework**

**Hypertension indicators:**

C1. Availability of anti-hypertensive and cardiovascular disease core medicines

C2. Availability of a functional device for measuring blood pressure

C3. Blood pressure control among people with hypertension, three months after treatment initiation (cohort-based)

C4. Blood pressure control among people with hypertension

O1. Screening for hypertension among adults as part of routine service

O2. Hypertension detection from opportunistic screening

O3. Treatment intensification due to uncontrolled blood pressure among patients under hypertension treatment

O4. Assessment for chronic kidney disease among people newly diagnosed with hypertension

O5. Assessment of cardiovascular disease risk among people with hypertension

O6. Stroke/heart attack among people with hypertension

O7. Availability of trained staff who are providing services for hypertension management

O8. Completeness and timelines of reporting of health facilities

O9. Facilities with supervision visit by district staff

O10. Loss to follow-up

**Diabetes indicators**

C1. Availability of diabetes core medicines

C2. Availability of plasma glucose testing

C3. Glycaemic control among people with diabetes, three months after diagnosis (cohort-based)

C4. Glycaemic control among people with diabetes

O1. Availability of HbA1c testing

O2. Treatment among people with diabetes

O3. Insulin therapy among people with diabetes requiring insulin

O4. Statin therapy among people with diabetes aged ≥40 years

O5. Treatment for chronic kidney disease among people with diabetes

O6. Treatment for elevated blood pressure among people with diabetes

O7. Assessment for diabetic chronic kidney disease  among people with diabetes

O8. Assessment for diabetic foot among people with diabetes

O9. Referral for retinopathy screening among people with diabetes

O10. Severe hypoglycaemia among people with diabetes

O11. Hyperglycaemic emergencies among people with diabetes

O12. Chronic kidney disease among people with diabetes

O13. Lower-limb amputation among people with diabetes

O14. Stroke/heart attack among people with diabetes

O15. Visual loss among people with diabetes

O16. Availability of trained staff who are providing services for diabetes management

O17. Completeness and timeliness of reporting of health facilities

O18. Facilities with supervision visit by district staff

O19. Loss to follow-up

**Chronic respiratory diseases indicators**

C1. Availability of asthma core medicines

C2. Availability of chronic obstructive pulmonary disease core medicines

C3. Asthma control, six months after treatment initiation (cohort-based)

C4. Chronic obstructive pulmonary disease control , six months after treatment initiation (cohort-based)

O1. Availability of peak flow meter

O2. Asthma diagnosis using peak flow measurement

O3. Chronic obstructive pulmonary disease diagnosis using peak flow measurement

O4. Treatment among people with asthma

O5. Treatment among people with chronic obstructive pulmonary disease

O6. Emergency visit among people with asthma

O7. Emergency visit among people with chronic obstructive pulmonary disease

O8. Availability of trained staff who are providing services for asthma/chronic obstructive pulmonary disease management

O9. Completeness and timeliness of reporting of health facilities

O10. Facilities with supervision visit by district staff

O11. Loss to follow-up

**Breast cancer indicators**

C1. Breast cancer early diagnosis/ screening by clinical breast examination among women aged 30–49 years

C2. Referral for diagnosis and management of suspected breast cancer

O1. Timeliness of breast cancer diagnosis initiation among women with abnormal clinical breast evaluation

O2. Timeliness of breast cancer diagnosis among women with breast cancer

O3. Referral for lymphedema evaluation for patients following breast cancer treatment

O4. Availability of trained staff who are providing clinical evaluation of breast abnormalities including the use of clinical breast examination

O5. Completeness and timeliness of reporting of health facilities

O6. Facilities with supervision visit by district staff

O7. Loss to follow-up

**Cervical cancer indicators**

C1. Availability of HPV testing

C2. Cervical cancer screening with high performance test among women aged 30–49 years

C3. Cervical cancer screening test positivity among women aged 30–49 years

O1. Availability of Pap smear testing

O2. Availability of visual inspection with acetic acid testing

O3. Cervical cancer screening among women aged 30–49 years

O4. Cervical cancer rescreening among women aged 30–49 years

O5. Cervical pre-cancer treatment

O6. Referral for diagnosis and management of suspected cervical cancer

O7. Availability of trained staff who are providing cervical cancer screening services

O8. Completeness and timeliness of reporting of health facilities

O9. Facilities with supervision visit by district staff

O10. Loss to follow-up

**Childhood cancers indicators**

C1. Early diagnosis among children with signs and symptoms associated with cancer

C2. Referral for diagnosis and management of suspected childhood cancer

O1. Availability of trained staff who are providing services for early diagnosis of childhood cancers

O2. Completeness and timeliness of reporting of health facilities

O3. Facilities with supervision visit by district staff

O4. Loss to follow-up

**General cancers indicators**

C1. Early diagnosis among people with signs and symptoms associated with cancer

C2. Referral for diagnosis and management of suspected cancer

O1. Availability of trained staff who are providing services for early diagnosis of cancers

O2. Completeness and timeliness of reporting of health facilities

O3. Facilities with supervision visit by district staff

O4. Loss to follow-up

**Supporting evidence**

**Supporting evidence for Hypertension and cardiovascular diseases indicators**

*Input/Process indicators*

**HC1: Availability of hypertension core medicines (Core)**

In a two-round Delphi study conducted for developing a framework for general practice from quality-of-care indicators used in 6 European countries, researchers, GPs and experts selected availability of core medicine as a core indicator ^1^.

**HC2: Availability of cardiovascular disease core medicines (Core)**

In a two-round Delphi study conducted to develop a framework for general practice from quality-of-care indicators used in 6 European countries, researchers, GPs and experts selected the availability of core medicine as a core indicator ^1^.

**HC3: Availability of a functional blood pressure measuring device (Core)**

In a set of indicators developed by the American College of Cardiology and American Heart Association Task Force, the availability of a blood pressure measuring device that allows precise measurement of blood pressure was chosen as a performance indicator ^2^.

*Output indicators*

**HO1: Assessment of cardiovascular disease risk (aged ≥40 years) among people aged over 40 years using WHO CVD risk charts (Optional)**

In a set of indicators developed by the American College of Cardiology and the American Heart Association Task Force, the assessment of CVD risk was chosen as a performance indicator ^2^. Moreover, in a set of indicators developed through a 4 stage Delphi in Canada, percentage of people with an average diastolic pressure of 90 mmHg or greater with risk factors including elevated systolic blood pressure, cigarette smoking, abnormal lipids, family history of premature cardiovascular disease, truncal obesity, sedentary lifestyle was chosen as a quality of care indicator ^3^, Moreover, in a Delphi study in which panels from 9 European countries chose indicator to assess quality of care of CVD diseases, risk assessment was included ^4^.

**HO2: Screening for hypertension among people aged 18 and over adults as part of routine service**

In a Delphi study in which panels from 9 European countries chose indicators to assess quality of care of CVD diseases, frequency of recording of BP was listed as a quality-of-care indicator ^4^. Moreover, in a study investigating 1,953 hypertensive patients, those who had optimal care were more likely to have their HTN under control and optimal care was determined from 27 indicators including measurement of systolic and diastolic BP at least once a year ^5^. Furthermore, in a modified Delphi study which was conducted to develop a quality measurement system for hypertension care in women of the West Coast in the United States, it was stated that SBP and DBP should be measured in adult women presenting for care ^6^. In a study that tested the feasibility of deriving comparative indicators in primary care practices in England, the percentage of the population who had their BP recorded during the past 5 years was included as an indicator ^7^.

**HO3: Hypertension detection from opportunistic screening (Optional)**

In a set of indicators developed through a four-stage Delphi in Canada, having a recorded diagnosis of HTN after high Blood Pressure (BP) in three distinct visits was counted as a quality of care indicator ^3^. Moreover, in a study in the United States, which investigated 1,953 hypertensive patients, those who had optimal care were more likely to have their HTN under control, and optimal care was determined from 28 indicators including documentation of HTN diagnosis after detection of high BP in three distinct visits ^5^. Additionally, in a modified Delphi study which provided 13 quality of care indicators for hypertension treatment in women of the West Coast of the United States, it was stated that hypertension diagnosis should be made after detection of high SDP and DBP in three distinct visits ^6^. In a study that tested the feasibility of deriving comparative indicators in primary care practices in England, the percentage of patients with HTN who had their BP recorded last year was listed as a quality of care indicator ^7^.

**HO4: Assessment for chronic kidney disease among people newly diagnosed with hypertension (Optional)**

In a four-step Delphi study which was conducted to develop a framework for quality-of-care assessment, the percentage of patients with a documented record of urinary protein excretion measurement was listed as an indicator ^3^. Furthermore, in a study on 1,953 hypertensive patients in the United States, those who received optimal care were more likely to have their HTN under control, and optimal care was determined from 29 indicators including measurement of serum creatinine and urine analysis ^5^. Moreover, the same indicator was also selected in a modified Delphi that was conducted to develop quality of care indicators for hypertension treatment in women of West Coast ^6^. In an observational study assessing quality of care in New England, serum electrolytes and creatinine were used as a quality-of-care indicator ^8^.

*Outcome indicators*

**HC4: Blood pressure control among people with hypertension (Core)**

In a four-stage Delphi study that developed a set of quality-of-care indicators in Canada, the percentage of hypertensive patients whose BP is at target was listed as a quality-of-care indicator ^3^. In a Delphi study in which panels from 9 European countries chose an indicator to assess the quality of care of CVD, control of HTN was selected as a quality of care indicator ^4^. In a study on 1,953 hypertensive individuals in the United States, the indicators that defined optimal care included various interventions to control different stages of HTN ^5^. The very same interventions were also provided in a set of 13 indicators which were developed to assess the quality of care in women of the West Coast of the United States ^6^. In an observational study assessing quality of care in New England, the percentage of patients with BP under 150/90 or less was counted as a quality of care indicator ^8^. In a study that tested the feasibility of deriving comparative indicators in primary care practices in England, they included the percentage of hypertensive patients who had their BP under control as an indicator ^7^.

**HO5: Blood pressure control among people with hypertension (follow-up) (Optional)**

In a four-step Delphi study conducted in Canada, percentage of patient visits with an action plan was provided as a quality-of-care indicator ^3^. In a Delphi study in which panels from 9 European countries chose indicators to assess the quality of care of CVD, it was stated that patients should be offered follow-up consultation within three months ^4^. In a study on 1,953 hypertensive individuals in the United States, the indicators which defined optimal care included annual check-up of hypertensive patients ^5^. Furthermore, in a modified Delphi study which developed 13 indicators to assess the quality of care of hypertension in women of the West Coast of the United States, annual measurement of BP was listed as one of the indicators ^6^. Moreover, in a study assessing quality of care in a country with universal health coverage (United States) annual visits of hypertensive patients were used to measure quality of care ^9^.

**Supporting evidence for diabetes mellitus indicators**

*Input/Process indicators*

**DC1: Availability of diabetes core medicines (Core)**

Based on WHO documentation about improving health outcomes of people with diabetes, the percentage of the population with type 1 diabetes with access to insulin and test strips, named availability of essential medications, was considered as a core quality of care metric ^10^.

**DC2: Availability of plasma glucose testing (Core)**

Based on WHO documentation about improving health outcomes of people with diabetes, the percentage of the population with type 1 diabetes with access to insulin and test strips, named availability of essential medications, was considered as a core quality of care metric ^10^.

**DC3: Availability of Hemoglobin A1c testing (Core)**

*Output indicators*

**DO1: Pharmacological treatment among people with diabetes (Optional)**

Delphi studies are the primary data source supporting Pharmacological treatment among people with diabetes ^11^ ^12^ ^13^ ^14^ ^15^ ^16^. A Delphi panel regarding developing a set of indicators for ambulatory diabetes care monitoring indicated the percentage of patients prescribed first-line drug class and second oral antihyperglycemic drug from different classes as an effective indicator ^12^. In another Delphi study for setting quality indicators for ambulatory care for the elderly with diabetes and related comorbidities, hypoglycemic drug use was an indicator with high consensus ^13^. In another Delphi study for developing type 2 diabetes quality indicators, which are extractable from electronic health records of general physicians, a variety of indicators about the pharmacological treatment of patients with diabetes have been selected ^14^. In another study regarding prescribing quality indicators of type 2 diabetes, receiving antihyperglycemic drugs in uncontrolled HbA1c or overweight patients with diabetes was mentioned multiple times with a few changes ^15^. In a randomized controlled study of patients with diabetes, for assessing the effect of benchmarking on improving the outcomes of diabetes, receiving glucose-lowering treatment was requested as a baseline characteristic of patients ^11^. In a review study of type 2 diabetes care guidelines in six European countries, patients who do not reach the HbA1c target should start or modify pharmacological treatment ^16^.

**DO2: Statin therapy among people with diabetes (Optional)**

Various study types provide the basis for the data that supports the implementation of statin therapy among individuals with diabetes ^10^ ^11^ ^13^ ^15^ ^16^. Based on WHO documentation about improving health outcomes of people with diabetes, statin use is a core care metric in diagnosed diabetes ^10^. In a randomized controlled study involving diabetes patients to evaluate the impact of benchmarking on enhancing diabetes outcomes, participants were required to have a history of receiving lipid-lowering treatment, which included statins, as one of their baseline characteristics ^11^. In a Delphi study aimed at establishing quality indicators for outpatient care for elderly individuals dealing with diabetes and related comorbidities, a strong consensus emerged in favor of recommending statin therapy for those who have diabetes, hypertension, and ischemic heart diseases ^13^. In another study focused on determining quality indicators for prescribing medications to individuals with type 2 diabetes, receiving statin in patients with high cardiovascular risk or aged ≤40 with a history of cardiovascular diseases was mentioned^15^.
In a review of type 2 diabetes care guidelines across six European countries, it was recommended that individuals who have cardiovascular diseases should be on lipid-lowering therapy ^16^.

**DO3: Pharmacological treatment for chronic kidney disease among people with diabetes (Optional)**

Different forms of studies contribute to the evidence supporting pharmacological treatment for chronic kidney disease among people with diabetes ^12^ ^14^ ^16^. In a review study of type 2 diabetes care guidelines in six European countries, the administration of ACE inhibitors to patients with microalbuminuria or proteinuria was considered a quality process indicator^16^. A Delphi expert panel focused on creating a set of metrics for monitoring outpatient diabetes care highlighted the proportion of diabetic patients with proteinuria who were prescribed ACE inhibitors or ARBs as an effective indicator ^12^. In another Delphi study focused on developing quality indicators for type 2 diabetes, which can be derived from the electronic health records of general physicians, the inclusion of pharmacological treatment for patients with diabetes and microalbuminuria was mentioned ^14^.

**DO4: Pharmacological treatment for hypertension among people with diabetes (Optional)**

Most of the data backing pharmacological treatment for hypertension among people with diabetes comes from Delphi studies ^17^ ^11^ ^12^ ^13^ ^14^ ^15^ ^16^. In a cross-sectional study, using metrics established by the American College of Cardiology/American Heart Association Task Force on Performance Measures, blood pressure control (including taking ≥2 antihypertensive medications) was used for assessing the quality of care among patients ^17^. In a randomized controlled study involving individuals with diabetes to evaluate the impact of benchmarking on diabetes outcomes, the inclusion of antihypertensive treatment was stipulated as a baseline patient characteristic ^11^. A Delphi panel tasked with creating indicators for monitoring diabetes care in outpatient settings identified the percentage of hypertensive patients not receiving antihypertensive treatment as a valuable indicator ^12^. In another Delphi study aimed at establishing quality benchmarks for outpatient care for elderly individuals with diabetes and other comorbidities, there was a strong consensus on using ACEI/ARB as an indicator ^13^. In another Delphi study focused on creating quality measures for type 2 diabetes, which can be derived from the electronic health records of general practitioners, a range of indicators related to the medication management of patients with diabetes and hypertension were chosen ^14^. In another study regarding prescribing quality indicators in type 2 diabetes patients, prescribing any antihypertensive medication in patients with diabetes and hypertension was discussed ^15^. In a review of guidelines for the care of individuals with type 2 diabetes in six European nations, there was an emphasis on ensuring that patients with hypertension receive suitable antihypertensive treatment ^16^.

**DO5: Assessment for diabetic chronic kidney disease among people with diabetes (Optional)**

Various study types provide the basis for the data that supports the assessment for diabetic chronic kidney disease among people with diabetes ^17^ ^18^ ^19^ ^11^ ^12^ ^20^ ^13^ ^14^ ^21^. In a cross-sectional investigation, the quality of care among patients was evaluated by assessing medical attention to nephropathy, which encompassed screening tests or clinical symptoms, using the metrics set forth by the American College of Cardiology/American Heart Association Task Force on Performance Measures ^17^. In a Delphi study focused on determining the quality of diabetes care in OECD countries, one of the process measures incorporated was the annual evaluation for microalbuminuria or the provision of medical attention to pre-existing nephropathy ^18^.

In a cross-sectional study aimed at assessing adherence to quality indicators for diabetes care in primary healthcare settings, urine tests were highlighted as indicators of the care process ^19^. In a randomized controlled study involving diabetes patients to evaluate the impact of benchmarking on enhancing diabetes outcomes, the inclusion of albuminuria was regarded as a baseline patient characteristic ^11^. A Delphi panel regarding developing a set of indicators for ambulatory diabetes care monitoring indicated an assessment of urinary microalbumin test and serum creatinine or estimated eGFR as risk factors of a care process that are effective ^12^. In a cross-sectional study to evaluate the appropriateness of electronic medical records as quality indicators, the percentage of patients with diabetes with a serum creatinine record or estimated GFR was mentioned ^20^. In another Delphi study aimed at defining quality benchmarks for outpatient care concerning elderly individuals with diabetes and concurrent health conditions, there was a notable level of agreement, both moderate and high, on using annual microalbumin testing and serum creatinine testing along with eGFR as key indicators ^13^. In another Delphi study for developing type 2 diabetes quality indicators, which are extractable from electronic health records of general physicians, a variety of indicators about nephropathy assessment have been selected ^14^. In a cross-sectional study for measuring the quality of care in patients with diabetes, annual urine tests were a process of care indicator ^21^.

**DO6: Assessment for diabetic foot among people with diabetes (Optional)**

Data supporting assessment for diabetic foot among people with diabetes are derived from various types of studies ^17^ ^18^ ^19^ ^11^ ^12^ ^14^ ^16^ ^21^. In a cross-sectional study, using metrics established by the American College of Cardiology/American Heart Association Task Force on Performance Measures, an annual foot exam was considered to assess the quality of care among patients ^17^. In a Delphi study regarding selecting the quality of diabetes care in OECD countries, receiving at least one foot examination annually was included as a process measure ^18^. In a cross-sectional study for measuring adherence rate to quality indicators of diabetes care in primary health care, diabetic foot screening was mentioned as a process indicator ^19^. In a randomized controlled study of patients with diabetes, for assessing the effect of benchmarking on improving the outcomes of diabetes, a foot examination was asked as a baseline characteristic of patients ^11^. A Delphi panel regarding developing a set of indicators for ambulatory diabetes care monitoring indicated foot examination as one of the care processes that are effective ^12^. In another Delphi study for developing type 2 diabetes quality indicators, which are extractable from electronic health records of general physicians, yearly foot examination has been considered an indicator ^14^. In a review study on type 2 diabetes care guidelines in six European countries, It has been mentioned that patients should have regular foot examinations, with intervals depending on the foot problem ^16^. In a cross-sectional study measuring the quality of care in patients with diabetes, annual foot examination was a process of care indicator ^21^.

**DO7: Referral for retinopathy screening among people with diabetes (Optional)**

Different forms of studies contribute to the evidence supporting Referral for retinopathy screening among people with diabetes ^17^ ^18^ ^19^ ^11^ ^12^ ^13^ ^14^ ^16^ ^21^. In a cross-sectional study that employed metrics established by the American College of Cardiology/American Heart Association Task Force on Performance Measures, an annual eye examination was considered as a means to assess the quality of care among patients^17^.In a Delphi study focused on determining the quality of diabetes care in OECD countries, one of the process measures included the requirement for patients to undergo either a dilated eye examination or an evaluation of retinal photography by an ophthalmologist or optometrist on an annual or biennial basis, depending on the risk of retinopathy^18^. A cross-sectional study, designed to measure adherence to quality indicators for diabetes care in primary healthcare settings, mentioned eye screening as a process indicator^19^. In a randomized controlled study involving patients with diabetes, conducted to assess the impact of benchmarking on diabetes outcomes, an eye examination was mandated as a baseline characteristic of patients ^11^. Furthermore, in a Delphi panel tasked with developing a set of indicators for monitoring ambulatory diabetes care, eye screening was identified as one of the effective care processes ^12^. In another Delphi study aimed at defining quality indicators for ambulatory care for elderly individuals with diabetes and related comorbidities, there was a high consensus in favor of conducting an eye examination every 1-2 years ^13^. In another Delphi study focused on establishing quality indicators for type 2 diabetes, which could be extracted from electronic health records maintained by general physicians, a variety of indicators related to eye examinations were selected ^14^. In a review study examining type 2 diabetes care guidelines across six European countries, it was highlighted that patients should undergo an eye risk factor classification and receive appropriate treatment in cases of retinopathy ^16^. In a cross-sectional study assessing the quality of care provided to patients with diabetes, a biennial eye examination performed by an ophthalmologist was designated as a process of care indicator ^21^.

*Outcome indicators*

**DC4: Glycaemic control among people with diabetes (Core)**

Data supporting glycaemic control among people with diabetes are derived from various types of studies ^10^ ^17^ ^22^ ^18^ ^19^ ^11^ ^12^ ^20^. According to a WHO documentation aimed at enhancing health outcomes for individuals with diabetes, the proportion of the diabetic population with well-controlled HbA1c levels, referred to as glycemic control, was regarded as a supplementary quality of care metric ^10^. In a cross-sectional study utilizing metrics established by the American College of Cardiology/American Heart Association Task Force on Performance Measures, it was recommended that patients undergo an annual assessment for glycemic control^17^. In a cross-sectional study assessing quality indicators in diabetes care, the percentage of diabetic patients with HbA1c levels below 7.5% within the past 12 months was considered as an indicator of treatment outcome^22^ In a Delphi study focused on determining the quality of diabetes care in OECD countries, the percentage of patients with poorly controlled diabetes was incorporated as a measure of treatment outcome ^18^. In a cross-sectional study aimed at evaluating adherence to quality indicators in diabetes care within primary healthcare settings, achieving an HbA1c level of 5.3% or lower was identified as an intermediate outcome indicator ^19^. In a randomized controlled study involving patients with diabetes, conducted to assess the impact of benchmarking on diabetes outcomes, the target HbA1c level was set at less than 7% ^11^. Furthermore, a Delphi panel tasked with establishing a set of indicators for monitoring ambulatory diabetes care indicated that the percentage of patients with suboptimal HbA1c levels was an effective indicator ^12^. In a cross-sectional study evaluating the appropriateness of electronic medical records as quality indicators, the percentage of diabetes patients whose HbA1c levels fell below various defined thresholds was taken into consideration ^20^.

**DO8: Glycaemic control among people with diabetes (follow-up) (Optional)**

Different forms of studies contribute to the evidence supporting glycaemic control among people with diabetes (follow-up) ^22^ ^19^ ^12^ ^20^ ^13^ ^14^ ^16^ ^21^ ^23^. In a cross-sectional study aimed at assessing quality indicators in diabetes care, the inclusion of patients with diabetes who had undergone at least one HbA1c measurement within the past 12 months was regarded as a process indicator ^22^. In a cross-sectional study focused on measuring adherence to quality indicators in diabetes care within primary healthcare, one of the process indicators was defined as conducting the HbA1c test twice annually^19^. A Delphi panel tasked with developing indicators for monitoring ambulatory diabetes care indicated that an effective metric involved patients receiving HbA1c tests within a 15-month timeframe ^12^. In a cross-sectional study that evaluated the suitability of electronic medical records as quality indicators, the assessment considered the percentage of diabetes patients whose HbA1C levels had fallen below various defined thresholds within the past 15 months ^20^. In another Delphi study aimed at establishing quality indicators for ambulatory care among elderly individuals with diabetes and related comorbidities, there was a high consensus on the inclusion of HbA1c testing every six months ^13^. In another Delphi study dedicated to developing quality indicators for type 2 diabetes, which could be extracted from electronic health records maintained by general physicians, one of the indicators involved measuring HbA1c levels at least once every six months ^14^. In a review study examining type 2 diabetes care guidelines across six European countries, it was emphasized that HbA1c concentration should be regularly monitored ^16^. In a cross-sectional study assessing the quality of care provided to patients with diabetes, one of the process of care indicators was having at least one HbA1c check annually ^21^. In a cohort study exploring various aspects of quality of care for individuals with type 1 diabetes, the study reported the percentage of patients who had undergone at least one HbA1c measurement during the year ^23^.

**DO9: Chronic kidney disease among people with diabetes (Optional)**

Based on WHO documentation about improving health outcomes of people with diabetes, the incidence of end-stage renal disease (ESRD) among the population with diagnosed diabetes, was considered as a complementary quality of care metric ^10^.

**DO10: Lower-limb amputation among people with diabetes (Optional)**

Based on WHO documentation about improving health outcomes of people with diabetes, the incidence of lower-extremity amputation (LEAs) among the population with diagnosed diabetes, was considered as a complementary quality of care metric ^10^. In a Delphi study for setting quality indicators for ambulatory care for the elderly with diabetes and related comorbidities, a lower-extremity amputation rate was an indicator with high consensus ^13^.

**DO11: Blindness among people with diabetes (Optional)**

In a Delphi study dedicated to evaluating quality benchmarks for outpatient care in elderly individuals dealing with diabetes and associated concurrent health issues, there was strong agreement on considering ocular complications resulting from diabetes as an indicator with high consensus ^13^.

**Supporting evidence for asthma and chronic obstructive pulmonary disease indicators**

*Input/Process indicators*

**AC1: Availability of asthma core medicines (Core)**

**AC2: Availability of chronic obstructive pulmonary disease core medicines (Core)**

**AO1: Availability of peak flow meter and mouthpiece (Optional)**

*Output indicators*

**AO2: Asthma diagnosis using peak flow measurement (Optional)**

Data supporting asthma diagnosis using peak flow measurement are derived from various types of studies ^24^ ^25^ ^26^. In an observational study aimed at identifying factors predicting the quality of asthma care in general practice, one of the quality indicators for primary asthma care involved the use of spirometry or peak flow measurement ^24^. In another study employing a modified RAND-appropriate method, a primary care performance indicator for asthma was the confirmation of the asthma diagnosis through spirometry, peak flow measurement, or the methacholine challenge test, with evidence levels ranging from II to IV ^25^. In another study that utilized validated performance indicators to assess the quality of asthma care in various primary care systems, two closely related indicators involved the utilization of spirometry for both diagnosis and ongoing monitoring ^26^.

**AO3: Chronic obstructive pulmonary disease diagnosis using peak flow measurement (Optional)**

In a study designed to assess the impact of sharing care quality information with patients on enhancing the care they receive, one of the quality of care indicators was the confirmation of COPD diagnosis through post-bronchodilator spirometry ^27^. Another study that explored the consequences of withdrawing incentives on the performance of general practices in managing COPD identified the use of spirometry for confirming the diagnosis as a quality of care indicator ^28^.

**AO4: Treatment among people with asthma (Optional)**

Evidence endorsing treatment among people with asthma is sourced from a variety of study types^25^ ^29^ ^26^ ^30^ ^31^ ^32^ ^33^ ^34^. In a study employing an adapted RAND-appropriate methodology, the use of controller medication served as a performance indicator for primary asthma care, supported by varying levels of evidence ^25^. In a review study, several indicators related to the administration of suitable treatments for patients with asthma were identified ^29^. In another study aimed at utilizing validated performance metrics to assess the quality of asthma care across different primary care systems, a diverse range of indicators pertaining to asthma medication prescription was considered ^26^. These indicators included different levels of patient adherence to inhaled corticosteroids and short-acting β2-agonists over the course of a year. In a cross-sectional study that evaluated adherence to national guidelines for quality of care, one of the assessed indicators involved the use of asthma medications ^30^. In a cohort study focused on evaluating the reliability of performance measurements for practice sites and physicians, ensuring the appropriate administration of asthma medications was among the process of care indicators ^31^. Another study examined the resilience of asthma quality indicators to data loss and included an indicator related to the receipt of asthma medication ^32^. Additionally, a study examined two quality indicators related to asthma care, specifically regarding the receipt of treatments, within Medicaid populations ^33^. In another study aimed at proposing quality of care metrics for electronic health records, one of the recommended indicators was the prescription of suitable medication for patients with asthma ^34^.

**AO5: Treatment among people with chronic obstructive pulmonary disease** **(Optional)**

In a cross-sectional study, quality of care in adherence to national guidelines was assessed, and medication use of COPD was among the evaluated indicators ^30^. In a study for assessing a national program's effect on COPD's clinical outcomes, treatment with different drug classes was evaluated ^35^.

*Outcome indicators*

**AC3: Asthma control (Core)**

Different forms of studies contribute to the evidence supporting asthma control^24 25 29^. In an observational study aimed at identifying factors that predict the quality of asthma care in general practice, there were indicators related to patient consultations and the assessment of asthma severity ^24^. In another study that utilized a modified RAND-appropriate approach, one of the primary care performance indicators for asthma involved determining the percentage of patients whose asthma symptom control had been assessed within the previous six months, supported by evidence levels III to IV ^25^. In a review study, achieving optimal asthma control among patients with asthma was highlighted as a measure of asthma care quality ^29^.

**AC4: Chronic obstructive pulmonary disease control (Core)**

**AO6: Emergency visit among people with asthma (Optional)**

Data supporting emergency visits among people with asthma are derived from various types of studies ^25^ ^26^ ^30^ ^36^. In another study using a modified RAND appropriate method, acute health service use, including emergency department or urgent care visits, was an asthma primary care performance indicator with level of evidence I-III,V ^25^. In another study aimed at using validated performance indicators to measure the quality of asthma care in different primary care systems, emergency department visits were mentioned ^26^. In a cross-sectional study, quality of care in adherence to national guidelines was assessed, and at least one emergency room visit due to asthma in 12 months was among the evaluated indicators ^30^. In a Delphi study aimed to develop indicators for childhood asthma, the percentage of patients who experienced emergency room visits due to an asthma attack and were reassessed within 72 hours by their doctor was one of the finally selected indicators ^36^.

**AO7: Emergency visit among people with chronic obstructive pulmonary disease (Optional)**

In a cross-sectional study that evaluated the quality of care in adherence to national guidelines, one of the assessed indicators was the occurrence of at least one emergency room visit for COPD within a 12-month period ^30^. In a study aimed at assessing the impact of a national program on the clinical outcomes of COPD, the frequency of annual admissions related to COPD was examined ^35^.

**Supporting evidence for breast cancer indicators**

*Output indicators*

**BC1: Clinical breast evaluation for early diagnosis of breast cancer among women aged 30–49 years with signs and/or symptoms associated with breast cancer (Core)**

In the Czech breast cancer screening program, the number of women recalled for further assessment was used as a measure to assess the quality of care ^37^. Furthermore, the Monitoring and Evaluation subgroup of the European Commission Initiative on Breast Cancer developed a set of indicators that included number of women recalled for further assessment who had positive screening examination ^38^.

**BC2: Timeliness of referral for breast cancer diagnosis among women aged 30–49 years with associated signs and /or symptoms of breast cancer who had suspicious findings from clinical breast evaluation (Core)**

In a two-step Delphi, consensus was achieved on Waiting time for referral to other health services to be a quality-of-care indicator ^39^. Moreover, in another study by the same group which included a literature review phase and a Delphi phase, waiting time for a referral to other health services was again selected as a quality-of-care indicator ^40^.

**BO1: Referral for mammography screening among women aged 50–69 years (Optional)**

In a study conducted to design a value stream map using EUSOMA, it was stated that the number of mammograms to be performed yearly should be more than 2000 ^41^. In another study investigating the quality of mammograms in Switzerland, the referral rate was included as a screening program performance indicator ^42^.

**BO2: Timeliness of breast cancer confirmatory diagnosis among women aged 30–49 years with suspicious findings from clinical breast evaluation (Optional)**

In a study investigating whether a community breast center could audit the quality of breast cancer care in collaboration with the National Consortium of Breast Centers (NCBC), the number of days between Screen to Diagnostic Mammogram and diagnostic mammogram to needle biopsy was used as a quality-of-care indicator ^43^.

**BO3: Timeliness of breast cancer treatment among women aged 30–49 years with suspicious findings from clinical breast evaluation (Optional)**

In a study investigating whether a community breast center could audit the quality of breast cancer care in collaboration with the National Consortium of Breast Centers (NCBC), the number of days between needle biopsy to surgery was selected as one of the quality of care indicators ^43^. Moreover, in the quality of care indicators developed by the Monitoring and Evaluation subgroup of the European Commission Initiative on Breast Cancer, the median number of days between screening and the start of first treatment was chosen as a quality of care indicator ^38^. Furthermore in an update from the EUSOMA working group, they stated that the Time interval from the date of first diagnostic examination within the breast center to the date of surgery or start of other treatment should be <6 weeks ^44^. The same indicator was provided in the first position paper of the EUSOMA which reported the results of a workshop in which twenty-four experts with diverse backgrounds came together to examine global literature and identify the primary process and outcome measures used for ensuring the quality of breast cancer care ^45^.

**Supporting evidence for cervical cancer indicators**

*Input/Process indicators*

**CC1: Availability of human papillomavirus testing (Core)**

**CO1: Availability of Pap smear testing (Optional)**

**CO2: Availability of visual inspection with acetic acid testing (Optional)**

*Output indicators*

**CC2: Cervical cancer screening with high performance test among women aged 30–49 years (Core)**

In a study focused on assessing the impact of a quality improvement program on cervical cancer screening, one of the quality indicators for screening in line with office protocols was the completion of both a Pap test and an HPV DNA test within a specified timeframe ^46^.

**CC3: Cervical cancer screening among women aged 30–49 years** **(Core)**

Various study types provide the basis for the data that supports cervical cancer screening among women aged 30–49 years^46 47 48 49 50 51^. In a study conducted to assess the influence of a quality improvement program on cervical cancer screening, one of the quality indicators was the adherence to office protocols for screening among all women aged 21 and older^46^ In a study focused on evaluating the performance indicators related to cervical screening, the outcomes of Pap smear tests were examined^47^. In another study, the results of cervical screening tests, specifically Pap smears, were utilized to calculate quality indicators ^48^. In a cross-sectional study aimed at evaluating the effectiveness of a quality indicator tool in cervical smear cytology, the results of cancer screening were employed ^49^. In another study that investigated cervical cancer screening policies and coverage in Europe, one of the metrics examined was the number of women covered by screening tests ^50^. In a study assessing the performance indicators of primary health centers, one of the measured factors was the frequency of eligible women who had undergone cervical cancer screening ^51^.

**CC4: Cervical cancer screening test positivity among women aged 30–49 years (Core)**

In a study examining the performance indicators of cervical screening, various positive outcomes of Pap smear tests were analyzed ^47^. In another study, the results from cervical screening were employed to calculate quality indicators for Pap smears, encompassing various positive conditions ^48^. In a cross-sectional study designed to assess the effectiveness of a quality indicator tool in cervical smear cytology, diverse positive smears were utilized to evaluate a range of indicators ^49^. In a study, the rate of positive test results served as a performance indicator ^52^. In a study focusing on process indicators within cervical cancer screening programs, diverse positive screening tests were documented ^53^.

**CO2: Cervical cancer rescreening among women aged 30–49 years (Optional)**

**CO3: Pre-invasive cervical disease treatment among women aged 30–49 years (Optional)**

**CO4: Timeliness of referral for cervical cancer diagnosis among women aged 30–49 years with suspicious findings from cervical cancer screening (Optional)**

In a study focusing on process indicators within cervical cancer screening programs, one of the indicators under consideration was the referral rate ^53^.

**Supporting evidence for childhood cancer indicators**

*Output indicators*

**ChC1: Clinical evaluation for early diagnosis of childhood cancer among children with signs and/or symptoms associated with cancer (Core)**

In a Delphi study aimed at evaluating the quality of a childhood cancer care delivery system, the Delphi panel endorsed the inclusion of the diagnosis of adolescent cancer in a pediatric center as an indicator ^54^.

**ChC2: Timeliness of referral for childhood cancer diagnosis among children with associated signs and/or symptoms of childhood cancer who had suspicious findings from clinical evaluation (Core)**

In a modified Delphi consensus study focusing on quality and capacity indicators for hospitalized pediatric oncology patients, the most prominent indicator within the service capacity domain was the prompt transfer of pediatric hematology-oncology patients from general wards to the pediatric intensive care unit ^55^.

**Supporting evidence for general cancer indicators**

*Output indicators*

**GC1: Clinical evaluation for early diagnosis of cancer among people with signs and/or symptoms associated with cancer (Core)**

**GC2: Timeliness of referral for cancer diagnosis among people with associated signs and/or symptoms of cancer who had suspicious findings from clinical evaluation (Core)**

**Supporting evidence for cross-cutting indicators**

*Input/Process indicators*

**IC1: Availability of trained staff (Core)**

 Data supporting the availability of trained staff are mainly derived from Delphi studies ^1 2 56 54 55 57^. In a Delphi study aimed at creating quality indicators for managing general practice in Europe, several indicators related to the accessibility and availability of well-qualified staff were discussed ^1^. In 2019, the American College of Cardiology/American Heart Association performance and quality measures for patients with hypertension guideline highlighted the importance of establishing infrastructure and personnel to address social determinants of health in patients and improving access to diverse care options ^2^. In another two-stage Delphi process aimed at validating predefined quality indicators for general practice, valid indicators related to access to physicians and staff qualifications were identified ^56^. In a Delphi study evaluating the quality of a childhood cancer care delivery system, the Delphi panel endorsed indicators related to having adequate multidisciplinary staff and ensuring access to experts ^54^.

In a modified Delphi consensus study focused on quality and capacity indicators for hospitalized pediatric oncology patients, the availability of physicians and nursing staff were identified as indicators within the personnel domain ^55^. In another study aiming to develop system performance indicators for adolescent and young adult cancer care, one of the indicators involved the presentation of patients at a multidisciplinary team meeting ^57^.

**IO1: Completeness and timeliness of reporting by health facilities (Optional)**

**IO2: Facilities receiving supervisory visit (Optional)**

*Output indicators*

**IC2: Loss to follow-up (Core)**

Various types of studies serve as the foundation for data that underscores the significance of follow-up, with all of them emphasizing the importance of follow-up itself rather than the occurrence of loss to follow-up ^24 25 27 26 44 45 58 59 47 57 60^. In an observational study focused on identifying predictors of the quality of asthma care in general practice, one of the indicators mentioned was the percentage of asthma patients who had undergone an asthma review ^24^. In another study employing a modified RAND-appropriate approach, primary care visits and the presence of routine care providers were identified as primary care performance indicators for asthma, supported by evidence level III ^25^. In a different study aimed at evaluating the impact of providing care quality data to patients on the improvement of care received, an annual review was considered one of the quality of care indicators ^27^. In another study targeting the assessment of validated performance indicators to gauge the quality of asthma care across various primary care systems, annual primary care visits were established as indicators related to outcomes ^26^. In studies concerning quality indicators in breast cancer care, the proper execution of follow-up procedures was highlighted ^44 45^. Another study that sought to evaluate the influence of hospital size on care processes following breast cancer identified the proportion of women who had received mammography after a history of breast cancer as an important follow-up indicator ^58^. In a study focused on developing and measuring a set of indicators for breast cancer, it was recommended to include annual mammography as part of the follow-up protocol ^59^. In a study assessing performance indicators for cervical screening, the evaluation included the proportion of patients with positive Pap smear tests who subsequently attended follow-up colposcopy appointments ^47^. In another study with the goal of establishing system performance indicators for cancer care among adolescents and young adults, receiving appropriate care during follow-up was emphasized ^57^. In another Delphi process aiming to define the quality of care metrics for outpatient pediatric oncology, the documentation of follow-up schedules and the receipt of appropriate treatment during follow-ups were determined to be important indicators ^60^.

**References**

1 Engels Y. Developing a framework of, and quality indicators for, general practice management in Europe. *Family Practice* 2005; **22**: 215–22.

2 Casey DE, Thomas RJ, Bhalla V, *et al.* 2019 AHA/ACC Clinical Performance and Quality Measures for Adults With High Blood Pressure. *Journal of the American College of Cardiology* 2019; **74**: 2661–706.

3 Burge FI, Bower K, Putnam W, Cox JL. Quality indicators for cardiovascular primary care. *Can J Cardiol* 2007; **23**: 383–8.

4 Campbell SM, Ludt S, Lieshout JV, *et al.* Quality indicators for the prevention and management of cardiovascular disease in primary care in nine European countries. *European Journal of Cardiovascular Prevention & Rehabilitation* 2008; **15**: 509–15.

5 Asch SM, McGlynn EA, Hiatt L, *et al.* Quality of care for hypertension in the United States. *BMC Cardiovasc Disord* 2005; **5**: 1.

6 Asch SM, Kerr EA, Lapuerta P, Law A, McGlynn EA. A New Approach for Measuring Quality of Care for Women With Hypertension. *Arch Intern Med* 2001; **161**: 1329.

7 McColl A. Clinical governance in primary care groups: the feasibility of deriving evidence-based performance indicators. *Quality in Health Care* 2000; **9**: 90–7.

8 Hammouche S, Holland R, Steel N. Does quality of care for hypertension in primary care vary with postcode area deprivation? An observational study. *BMC Health Serv Res* 2011; **11**: 297.

9 Collet T-H, Salamin S, Zimmerli L, *et al.* The quality of primary care in a country with universal health care coverage. *J Gen Intern Med* 2011; **26**: 724–30.

10 Improving Health Outcomes of People with Diabetes Mellitus. https://www.who.int/publications/m/item/improving-health-outcomes-of-people-with-diabetes-mellitus (accessed June 4, 2023).

11 Hermans MP, Brotons C, Elisaf M, Michel G, Muls E, Nobels F. Optimal type 2 diabetes mellitus management: the randomised controlled OPTIMISE benchmarking study: baseline results from six European countries. *Eur J Prev Cardiol* 2013; **20**: 1095–105.

12 Mukerji G, Halperin I, Hunter K, *et al.* Developing a set of indicators to monitor quality in ambulatory diabetes care using a modified Delphi panel process. *Int J Qual Health Care* 2018; **30**: 65–74.

13 Petrosyan Y, Barnsley JM, Kuluski K, Liu B, Wodchis WP. Quality indicators for ambulatory care for older adults with diabetes and comorbid conditions: A Delphi study. *PLoS One* 2018; **13**: e0208888.

14 Van den Bulck SA, Vankrunkelsven P, Goderis G, *et al.* Development of quality indicators for type 2 diabetes, extractable from the electronic health record of the general physician. A rand-modified Delphi method. *Prim Care Diabetes* 2020; **14**: 75–84.

15 Martirosyan L, Braspenning J, Denig P, *et al.* Prescribing quality indicators of type 2 diabetes mellitus ambulatory care. *Qual Saf Health Care* 2008; **17**: 318–23.

16 Wens J, Dirven K, Mathieu C, Paulus D, Van Royen P. Quality indicators for type-2 diabetes care in practice guidelines: an example from six European countries. *Prim Care Diabetes* 2007; **1**: 17–23.

17 Arnold SV, Goyal A, Inzucchi SE, *et al.* Quality of Care of the Initial Patient Cohort of the Diabetes Collaborative Registry®. *J Am Heart Assoc* 2017; **6**: e005999.

18 Nicolucci A, Greenfield S, Mattke S. Selecting indicators for the quality of diabetes care at the health systems level in OECD countries. *Int J Qual Health Care* 2006; **18 Suppl 1**: 26–30.

19 Al-Ubaidi BA, Al-Khadraji MA, Al-Hermi B. Measuring adherence rate to quality indicators for diabetes care identified by primary health care in Bahrain. *Saudi Med J* 2014; **35**: 975–80.

20 Djalali S, Frei A, Tandjung R, Baltensperger A, Rosemann T. Swiss quality and outcomes framework: quality indicators for diabetes management in Swiss primary care based on electronic medical records. *Gerontology* 2014; **60**: 263–73.

21 Zuercher E, Diatta ID, Burnand B, Peytremann-Bridevaux I. Health literacy and quality of care of patients with diabetes: a cross-sectional analysis. *Primary care diabetes* 2017; **11**: 233–40.

22 Meier R, Valeri F, Senn O, Rosemann T, Chmiel C. Quality performance and associated factors in Swiss diabetes care - A cross-sectional study. *PLoS One* 2020; **15**: e0232686.

23 Bruno G, Pagano E, Rossi E, *et al.* Incidence, prevalence, costs and quality of care of type 1 diabetes in Italy, age 0–29 years: The population-based CINECA-SID ARNO Observatory, 2002–2012. *Nutrition, Metabolism and Cardiovascular Diseases* 2016; **26**: 1104–11.

24 Abdelhamid AS, Maisey S, Steel N. Predictors of the quality of care for asthma in general practice: an observational study. *Family Practice* 2010; **27**: 186–91.

25 To T, Guttmann A, Lougheed MD, *et al.* Evidence-based performance indicators of primary care for asthma: a modified RAND Appropriateness Method. *International Journal for Quality in Health Care* 2010; **22**: 476–85.

26 To T, Guan J, Zhu J, *et al.* Quality of asthma care under different primary care models in Canada: a population-based study. *BMC Fam Pract* 2015; **16**: 19.

27 Roberts CM, Gungor G, Parker M, Craig J, Mountford J. Impact of a patient-specific co-designed COPD care scorecard on COPD care quality: a quasi-experimental study. *npj Prim Care Resp Med* 2015; **25**: 15017.

28 Kontopantelis E, Springate D, Reeves D, Ashcroft DM, Valderas JM, Doran T. Withdrawing performance indicators: retrospective analysis of general practice performance under UK Quality and Outcomes Framework. *BMJ* 2014; **348**: g330–g330.

29 Herman E, Beavers S, Hamlin B, Thaker K. Is It Time for a Patient-Centered Quality Measure of Asthma Control? *The Journal of Allergy and Clinical Immunology: In Practice* 2019; **7**: 1771–7.

30 Neugaard BI, Priest JL, Burch SP, Cantrell CR, Foulis PR. Quality of Care for Veterans with Chronic Diseases: Performance on Quality Indicators, Medication Use and Adherence, and Health Care Utilization. *Population Health Management* 2011; **14**: 99–106.

31 Sequist TD, Schneider EC, Li A, Rogers WH, Safran DG. Reliability of Medical Group and Physician Performance Measurement in the Primary Care Setting. *Medical Care* 2011; **49**: 126–31.

32 Stuart B, Singhal PK, Magder LS, Zuckerman IH. How Robust Are Health Plan Quality Indicators to Data Loss? A Monte Carlo Simulation Study of Pediatric Asthma Treatment: How Robust Are Health Plan Quality Indicators to Data Loss? *Health Services Research* 2003; **38**: 1547–62.

33 Samnaliev M, Baxter JD, Clark RE. Comparative Evaluation of Two Asthma Care Quality Measures Among Medicaid Beneficiaries. *Chest* 2009; **135**: 1193–6.

34 Kern LM, Dhopeshwarkar R, Barrón Y, Wilcox A, Pincus H, Kaushal R. Measuring the Effects of Health Information Technology on Quality of Care: A Novel Set of Proposed Metrics for Electronic Quality Reporting. *The Joint Commission Journal on Quality and Patient Safety* 2009; **35**: 359-AP2.

35 Park HJ, Kim S-R, Kim S, *et al.* Influence of government-driven quality assessment program on patients with chronic obstructive pulmonary disease. *Respir Res* 2021; **22**: 87.

36 Ruiz-Canela-Cáceres J, Aquino-Llinares N, Sánchez-Diaz JM, García-Gestoso ML, de Jaime-Revuelta ME, Praena-Crespo M. Indicators for childhood asthma in Spain, using the Rand method. *Allergologia et Immunopathologia* 2015; **43**: 147–56.

37 Majek O, Danes J, Skovajsova M, *et al.* Breast cancer screening in the Czech Republic: time trends in performance indicators during the first seven years of the organised programme. *BMC public health* 2011; **11**: 1–13.

38 Muratov S, Canelo-Aybar C, Tarride J-E, *et al.* Monitoring and evaluation of breast cancer screening programmes: selecting candidate performance indicators. *BMC cancer* 2020; **20**: 1–10.

39 Benito L, de la Cueva Ariza L, Delgado-Hito P, Martinez Momblan MA, Romero García M, García M. Identifying Coordination and Continuity of Care Indicators for Population-Based Cancer Screening Programs: A Delphi Study. *Nursing Research* 2018; **67**: 411.

40 Benito L, Espinosa J, Binefa G, *et al.* Population-based Cancer Screening: Measurement of Coordination and Continuity of Care. *Cancer Nursing* 2018; **41**: E1.

41 Camgoz Akdag H, Canturk NZ. Improvement of Breast Cancer Patient Pathway Using EUSOMA Standards and European Guidelines. *Chirurgia* 2017; **112**. DOI:10.21614/chirurgia.112.4.449.

42 Bulliard J-L, Ducros C, Dayer E, Arzel B, Levi F. Variation in performance in low-volume mammography screening programmes: Experience from Switzerland. *Cancer Epidemiology* 2011; **35**: 293–7.

43 Landercasper J, Ellis RL, Mathiason MA, *et al.* A community breast center report card determined by participation in the national quality measures for breast centers program. *The breast journal* 2010; **16**: 472–80.

44 Biganzoli L, Marotti L, Hart CD, *et al.* Quality indicators in breast cancer care: An update from the EUSOMA working group. *European Journal of Cancer* 2017; **86**: 59–81.

45 Del Turco MR, Ponti A, Bick U, *et al.* Quality indicators in breast cancer care. *European journal of cancer* 2010; **46**: 2344–56.

46 Hills RL, Kulbok PA, Clark M. Evaluating a Quality Improvement Program for Cervical Cancer Screening at an Urban Safety Net Clinic. *Health Promotion Practice* 2015; **16**: 631–41.

47 Bucchi L, Falcini F, Schincaglia P, *et al.* Performance indicators of organized cervical screening in Romagna (Italy): *European Journal of Cancer Prevention* 2003; **12**: 223–8.

48 Costa RFA, Longatto-Filho A, de Lima Vazquez F, Pinheiro C, Zeferino LC, Fregnani JHTG. The Quality of Pap Smears from the Brazilian Cervical Cancer Screening Program According to the Human Development Index. *Cancer Prev Res* 2020; **13**: 299–308.

49 Magalhães JC, Ázara CZS, Tavares SB do N, Manrique EJC, Amaral RG. Impact of Implementing 100% Rapid Review as a Quality Control Tool in Cervical Cytology. *Acta Cytologica* 2018; **62**: 115–20.

50 Anttila A, von Karsa L, Aasmaa A, *et al.* Cervical cancer screening policies and coverage in Europe. *European Journal of Cancer* 2009; **45**: 2649–58.

51 Naumovic T, Jovanovic V, Ilic D, Rakic U, Mirkov D, Perisic Z. Performance indicators collected from primary health centres included in organised cervical cancer screening programme in the Republic of Serbia. *J BUON* 2015; **20**: 842–6.

52 Tobias A, Amaral R, Diniz E, Carneiro C. Quality Indicators of Cervical Cytopathology Tests in the Public Service in Minas Gerais, Brazil. *Rev Bras Ginecol Obstet* 2016; **38**: 065–70.

53 Ronco G, Giubilato P, Naldoni C, *et al.* Extension of organised cervical cancer screening programmes in Italy and their process indicators. *Epidemiol Prev* 2007; **31**: 33–47.

54 Bradley NME, Robinson PD, Greenberg ML, *et al.* Measuring the Quality of a Childhood Cancer Care Delivery System: Assessing Stakeholder Agreement. *Value in Health* 2013; **16**: 639–46.

55 Arias AV, Garza M, Murthy S, *et al.* Quality and capacity indicators for hospitalized pediatric oncology patients with critical illness: A modified delphi consensus. *Cancer Med* 2020; **9**: 6984–95.

56 Campbell SM, Roland MO, Quayle JA, Buetow SA, Shekelle PG. Quality indicators for general practice: which ones can general practitioners and health authority managers agree are important and how useful are they? *Journal of Public Health* 1998; **20**: 414–21.

57 Rae CS, Pole JD, Gupta S, *et al.* Development of System Performance Indicators for Adolescent and Young Adult Cancer Care and Control in Canada. *Value in Health* 2020; **23**: 74–88.

58 Vrijens F, Stordeur S, Beirens K, Devriese S, Van Eycken E, Vlayen J. Effect of hospital volume on processes of care and 5-year survival after breast cancer: A population-based study on 25 000 women. *The Breast* 2012; **21**: 261–6.

59 Stordeur S, Vrijens F, Devriese S, Beirens K, Van Eycken E, Vlayen J. Developing and measuring a set of process and outcome indicators for breast cancer. *The Breast* 2012; **21**: 253–60.

60 Teichman J, Punnett A, Gupta S. Development of Quality Metrics to Evaluate Pediatric Hematologic Oncology Care in the Outpatient Setting. *Journal of Pediatric Hematology/Oncology* 2017; **39**: 90–6.
